# Flavobacterial exudates disrupt cell cycle progression and metabolism of the diatom *Thalassiosira pseudonana*

**DOI:** 10.1038/s41396-022-01313-9

**Published:** 2022-09-14

**Authors:** Zinka Bartolek, Shiri Graff van Creveld, Sacha Coesel, Kelsy R. Cain, Megan Schatz, Rhonda Morales, E. Virginia Armbrust

**Affiliations:** School of Oceanography, University of Washington, Seattle, WA 98195 USA

**Keywords:** Transcriptomics, Microbial biooceanography

## Abstract

Phytoplankton and bacteria form the base of marine ecosystems and their interactions drive global biogeochemical cycles. The effects of bacteria and bacteria-produced compounds on diatoms range from synergistic to pathogenic and can affect the physiology and transcriptional patterns of the interacting diatom. Here, we investigate physiological and transcriptional changes in the marine diatom *Thalassiosira pseudonana* induced by extracellular metabolites of a known antagonistic bacterium *Croceibacter atlanticus*. Mono-cultures of *C. atlanticus* released compounds that inhibited diatom cell division and elicited a distinctive morphology of enlarged cells with increased chloroplast content and enlarged nuclei, similar to what was previously observed when the diatom was co-cultured with live bacteria. The extracellular *C. atlanticus* metabolites induced transcriptional changes in diatom pathways that include recognition and signaling pathways, cell cycle regulation, carbohydrate and amino acid production, as well as cell wall stability. Phenotypic analysis showed a disruption in the diatom cell cycle progression and an increase in both intra- and extracellular carbohydrates in diatom cultures after bacterial exudate treatment. The transcriptional changes and corresponding phenotypes suggest that extracellular bacterial metabolites, produced independently of direct bacterial-diatom interaction, may modulate diatom metabolism in ways that support bacterial growth.

## Introduction

Diatoms are photosynthetic eukaryotes responsible for up to 20% of annual photosynthesis on Earth [1, 2]. They serve as the base of many marine food webs and can form dense blooms that reach relatively high cell abundances. Diatoms have co-existed and co-evolved with bacteria for millions of years, ultimately developing close interactions that facilitate the exchange of nutrients and metabolites [3]. These interactions are hypothesized to occur within the phycosphere, a diffusive boundary layer that surrounds individual phytoplankton cells. Here, mass transport is limited to diffusion and is not impacted by turbulence [4, 5]. Within this microenvironment, nutrient and fixed carbon concentrations can exceed background levels by orders of magnitude, and bacterial growth can be stimulated by extracellular metabolites of the diatom [6]. The phycosphere also contains enriched concentrations of signaling compounds and secondary metabolites that modify the behavior of the host diatom and associated bacteria [7].

Diatoms and bacteria display pathogenic and mutualistic interactions, some of them obligatory [3, 5, 7]. When co-cultured with diatoms, bacteria can influence diatom growth rate, metabolism, and cell cycle progression [8–12]. Fundamentally, the interactions between diatoms and bacteria are based on various metabolic exchanges. In mutualistic interactions, bacteria provide micronutrients necessary for diatom growth, such as vitamin B_12_ or bioavailable iron in the form of siderophores, while the diatom supplies the bacteria with readily accessible carbon and sulfur sources necessary for bacterial growth [5, 11, 13]. Interactions between diatoms and bacteria are often species-, and even strain-specific, where the interacting bacteria are adapted to a relatively narrow range of compounds secreted by the diatom, resulting in distinct bacterial communities that are consistent across strains and time [14–17]. Conversely, diatoms can shape the composition of their bacterial communities through excreted metabolites to select for bacterial communities that are beneficial to the diatom and to prevent the establishment of potentially harmful bacteria [18–20]. Additionally, phytoplankton exudates can influence the production of the bacterial exo-metabolome [21].

Algicidal bacteria generally belong to the phyla of *Bacteroidetes*, *Gammaproteobacteria*, and *Alphaproteobacteria*, and interact with diatoms through direct contact, close proximity to the diatom cell, or through production of excreted algicides [7]. Bacterial attachment to other phytoplankton such as dinoflagellates and coccolithophorids has also been described [22, 23]. Algicide production by bacteria often depends on the high nutrient availability found in the phycosphere, and their structure and mode of action can range from small metabolites with specific cellular targets to large enzymes catalyzing the change of numerous processes in the cell [7]. For example, bacterial quorum sensing compounds can have antagonistic effects on diatom growth and transcriptional changes, with long chain N-acyl homoserine lactones inhibiting growth of *Seminavis robusta* and inducing changes in lipid metabolism and cell cycle progression [24, 25]. However, the activity of algicidal compounds produced by bacteria in a contact-independent manner is understudied and not well understood.

Using a bacterial consortium previously isolated from the diatom *Pseudo-nitzschia multiseries*, Amin et al. demonstrated the effect of various single strain isolates on diatom physiology through co-culture experiments with different diatom species [11]. The isolated flavobacterium *Croceibacter atlanticus* (SA60) exhibited an inhibitory effect on several different diatoms including the model *Thalassiosira pseudonana* [10]. In co-culture, live *C. atlanticus* attached directly to the surface of diatom cells and inhibited *T. pseudonana* cell division and induced enlarged cells with multiple plastids [10]. Here, we asked whether a similar diatom morphology could be elicited by *C. atlanticus* bacterial exudates in bacterial-free culturing conditions. We investigated the effect of extracellular bacterial metabolites produced by mono-cultures of *C. atlanticus* on the growth, morphology, and transcriptional profiles of the diatom *T. pseudonana*. Resulting changes in cell growth and transcriptional profiles of diatoms exposed to *C. atlanticus* extracellular metabolites suggest a disruption of cell cycle-related processes and an enhancement of carbohydrate and amino acid metabolism, independent of bacterial attachment. Phenotyping of treated diatom cultures confirmed cell cycle disruption and increased carbohydrate concentrations in the media of treated diatom cells. This indicates that metabolites produced and released by *C. atlanticus* bacteria that were not previously exposed to diatoms can modulate the transcriptional profile, physiology, and phycosphere composition of diatoms to potentially enhance bacterial growth.

## Methods

### *C. atlanticus* filtrate and H_2_O_2_ treatment of *T. pseudonana* cells

Glycerol stocks of *C. atlanticus* SA60 NCMA B36 (National Center for Marine Algae and Microbiota, NCMA, East Botany, ME, USA) isolated from *Pseudo-nitzschia multiseries* CLNN-17 [11] were streaked on marine agar plates (marine broth (MB) with 1.5% w/v agar) and grown at 30 °C for 3-4 days. Single colonies were grown overnight in MB with shaking at 30 °C until an OD_600_ (NanoDrop One Microvolume UV-Vis Spectrophotometer OD_600_) of ~0.3 was reached. Cells were inoculated into synthetic seawater medium Aquil (National Center for Marine Algae and Microbiota (NCMA)) with 5% MB (v/v), grown with shaking at 30 °C until the exponentially growing cells reached OD_600_ = 0.3 (4 × 10^8^ cells/ml), filtered twice through a 0.22 µm glass fiber filter; and the resulting filtrate was collected and checked microscopically for bacterial contamination by staining with SYBR Green (1:10000) for 20 min. Additionally, 500 µl of filtrate was inoculated into 10 ml of MB, incubated at room temperature for 5 days and inspected visually for bacterial contaminants. This sterile filtrate was stored in the dark at 4 °C prior to use.

To measure the effect of bacterial-free *C. atlanticus* filtrate on *T. pseudonana* cell growth, the filtrate was added at different concentrations (1.6, 3.3, 13, 33, 66; and 100% v/v filtrate to L1 + Si medium) to triplicate treatment flasks of 5000 cells/ml *T. pseudonana* CCMP 1335 (obtained from NCMA) that had been maintained as a semicontinuous batch culture in 30 ml L1 + Si medium (NCMA) [26] at 20 °C in a 16:8 h light:dark cycle at ~ 160 µmol photons m^−2^ sec^−1^. Control *T. pseudonana* cultures were treated with corresponding volumes of filtered Aquil with 5% v/v MB (the bacterial growth media). A more comprehensive time course experiment was conducted with control cultures and 33% v/v of bacterial-free *C. atlanticus* filtrate in a final volume of 2 L. At 7 h into the light period of each day of the 8-day experiment, in vivo chlorophyll *a* fluorescence (Turner Designs 10-AU fluorometer) was measured, and samples were collected to determine cell abundance (Guava easyCyte Plus Flow Cytometry System) and photochemical yield of photosystem II (Fv/Fm) after a 15 min dark acclimation (Phyto-PAM fluorometer). Samples for microscopy (2 ml) and for cell cycle analysis (2 ml) were taken daily, incubated for 20 min with 2% v/v glutaraldehyde, flash frozen in liquid nitrogen, and stored at −80 °C prior to analysis. Samples (200 ml) for RNA extraction were collected from each replicate flask at three timepoints: 24 h, 72 h, and 120 h after the start of the experiment by filtering diatom cells onto 0.2 µm polycarbonate membrane filters, flash freezing in liquid nitrogen, and storing the filters at −80 °C prior to analysis. In a separate experiment using the same treatment and control conditions in a final volume of 30 ml, triplicate samples were collected for diatom cell cycle analysis every 2 h for 12 h, fixed with glutaraldehyde, flash frozen in liquid nitrogen and stored at −80 °C.

Exponentially growing *T. pseudonana* cells (f/2+Si media at 20 °C in a 16:8 h light:dark cycle at ~ 160 µmol photons m^−2^ sec^−1^) were treated in triplicate with 0 µM or 200 µM of hydrogen peroxide. Two hours after treatment, 500 ml of cultures were harvested for RNA extraction as described above.

### Cell morphology measurements and imaging

*T. pseudonana* cells were imaged via epifluorescence microscopy (470/40 nm blue excitation 515 nm long-pass emission filter) and brightfield imaging. To obtain measurements of nuclear area and chloroplast area, 1 ml of glutaraldehyde-preserved diatom cells was concentrated onto black 0.2 µm pore size polycarbonate filters, the DNA of cells collected on the filter was stained using a SYBR Green I stain, and the filter subsequently attached to microscope slides, as described in van Tol et al., 2017 [10]. Fluorescent images at 60x magnification were taken (Nikon eclipse 80i microscope) and processed using ImageJ [27] software. Nucleus and chloroplast areas were obtained by splitting the 60x fluorescent images into three channels (RGB), and the wand tool was used to select nuclei (green channel) and chlorophyll fluorescence (red channel). The Kolmogorov-Smirnov test was used to test for significant differences (*p* < 0.05) in distributions of control compared to treated cells at each time point. To obtain measurements of cell area, 1 ml of glutaraldehyde-preserved diatoms cells was stained with SYBR Green (1:10000), incubated for 20 min at room temperature, spun down at 10000 rpm for 1 min, and the supernatant discarded for a final volume of 10 µl. Resulting concentrated diatom cells were mounted onto transparent slides, and fluorescent and brightfield images at 20x magnification were taken (Leica DMi8 microscope). Cell area and maximum and minimum cell diameter measurements were obtained from 20x brightfield images using the ImageJ [27] wand tool and straight-line selection tool. Analysis was restricted to in-focus cells entirely within the camera frame.

### Flow cytometry

Samples for flow cytometry were stained with SYBR Green I (1:10000), incubated for 20 min, and run on a BD Influx flow cytometer (BD, Franklin Lakes, NJ, USA) (488 nm laser; emission 530 nm, 40 nm band pass) on a linear scale with at least 10,000 *T. pseudonana* cells analyzed per sample [10]. *T. pseudonana* cells were gated using the FCSplankton package in R (https://github.com/fribalet/FCSplankton). SYBR Green I-stained cells were manually gated on forward scatter and 530/40 nm emission using the set_gating_params() function. Forward scatter distributions, as a proxy for cell size, were normalized to 1 μm beads (Molecular Probes, Eugene, OR, USA) that were added to each sample as an internal standard. Contrary to control cultures, the G1, S, and G2 populations of the filtrate-treated cultures did not follow a Gaussian distribution, precluding quantitative cell cycle decomposition of these samples. To compare the distributions of the SYBR-stained control and filtrate-treated cells, the distributions were aligned to the mode of the G1 peak. Cell cycle stage boundaries were drawn based on the signal of the control cultures, allowing for qualitative comparison of the G1, S, and G2 population distributions between control and filtrate treated cultures.

### Carbohydrate content analysis

Carbohydrate content was determined via the phenol-sulfuric acid assay as described in DuBois et al. 1956 [28]. *T. pseudonana* cells (initial concentration 1 × 10^5^cells/ml) were transferred in triplicate into either control media (Aquil + 5% MB) or undiluted bacterial-free *C. atlanticus* filtrate (30 ml total volume) and 2 ml samples were collected for total (dissolved + particulate) and dissolved carbohydrate content at 24 h and 48 h. Samples for dissolved carbohydrates were filtered through a 0.4 µm filter. Both the resulting filtrate and samples for total carbohydrates were stored at −80 °C. To control for carbohydrates introduced by the bacterial filtrate itself, samples of bacterial-free *C. atlanticus* filtrate were also measured. For each replicate, 250 µl sample was combined with 250 µl of 5% phenol in DI water and 1250 µl of concentrated sulfuric acid, gently mixed, and incubated for 20 min at room temperature. Absorbance at 485 nm was measured and compared to a dilution series of glucose standards. To obtain the final carbohydrate content of treated cells, the carbohydrate content of the *C. atlanticus* filtrate was subtracted from measurements of total and dissolved fractions of treated cells. Particulate carbohydrate content was calculated as the difference between total and dissolved fractions. The students *t*-test was used to test for significance (*p* < 0.05) between control and treated cells at each time point.

### *T. pseudonana* transcriptome sequencing and analysis

The *T. pseudonana* transcriptome processing pipeline of control and filtrated-treated cultures (Fig. S1) included RNA extraction, sequencing, and quantification (blue boxes); identification of differentially transcribed genes and genes that were co-transcribed (yellow boxes); and gene enrichment analyses (green boxes) to identify subsets of genes for further analyses. The code applied for this analysis is shared on GitHub page (https://github.com/armbrustlab/Thaps_Catl) and all data are deposited on Zenodo (10.5281/zenodo.6672614).

Total RNA was extracted from 0.2 µm polycarbonate membrane filters with the Zymo Direct-zol RNA MiniPrep Plus kit. Poly-A-selection, library preparation and sequencing were performed at the Northwest Genomics Center (University of Washington) with NextSeq (Illumina). Sequence reads were trimmed using Trimmomatic 0.39 [29], run in paired-end mode with cut adaptor and other Illumina-specific sequences (ILLUMINACLIP) set to TruSeq3-PE.fa:2:30:10:1:true, Leading and Trailing thresholds of 25, a sliding window trimming approach (SLIDINGWINDOW) of 4:15, an average quality level (AVGQUAL) of 20, and a minimum length (MINLEN) of 60. Reads were mapped to the genome of *T. pseudonana* (Thaps3 FilteredModels2) using Hisat2-2.1.0. We calculated the number of *T. pseudonana* reads that aligned to the gene models from resulting SAM alignment files with aligned sequences used in subsequent analyses and normalized using transcripts per million (TPM).

The edgeR pipeline [30] and Weighted Gene Co-expression Network Analysis (WCGNA) [31] were used in parallel to identify the transcriptional changes in response to treatment with the bacterial filtrate (yellow boxes, Fig. S1). EdgeR was used to detect differential expression of log_2_ transformed transcript levels based on pairwise comparisons between the control samples and the samples treated with bacterial-free *C. atlanticus* filtrate for each sampled time point (24 h, 72 h, and 120 h). A generalized linear model (GLM) quasi-likelihood F-test (QLTF) was used to test for significant differential expression (*p* < 0.01 and false discovery rate (FDR) < 0.05). Significantly differentially expressed genes with |logFC | > 1 were used for downstream analysis.

Signed WCGNA was used to identify modules of co-expressed genes using both control and treatment transcriptomes with log_2_ transformed transcripts per million (TPM) as input abundance data; those genes with transcript abundance below the TPM median in greater than 25% of samples were removed from analysis (Fig. S2A; Fig. S1). Two outlier samples (t24_cont_3 and t72_cont_72) were removed from further network analysis as recommended [31], based on a branch height > 60 in the sample clustering dendrogram of Euclidean distance between samples (Fig. S2B). To identify modules of groups of genes with similar expression patterns, a network of gene co-expression was constructed by making a weighted adjacency matrix that was then transformed into a topological overlay matrix (TOM) to estimate connectivity in the network. Average linkage hierarchical clustering with a soft-threshold power=18 (Fig. S2C) was used to construct the clustering tree structure of the TOM (Fig. S3A) [31]. Modules were assigned based on a dynamic tree cut method (minimum number of genes per module=30; deep split=1), and similar modules were merged (cut height threshold=0.25) (Fig. S3). Each module was correlated to different parameters measured during the experiment to identify modules with strongest correlation to treatment with the bacterial filtrate. Modules with a Pearson’s correlation >0.6 to *C. atlanticus* filtrate treatment were selected based on module eigengenes (MEs) and module significance >0.5 (average gene significance for each module, where gene significance is the log10 transformed *p* value from the linear regression between gene expression and *C. atlanticus* filtrate treatment) (Fig. S4).

Functional enrichment analysis (green boxes, Fig. S1) was performed with topGO [32] using a custom gene ontology (GO) annotation file (Joint Genome Institute) and a classic Fisher test (*p* < 0.01). Functional enrichment analysis was performed on each WGCNA module, as well as on the differentially expressed genes at each of the three timepoints identified by edgeR (Table S1). Enrichment analysis of GO Biological Process (BP) terms were visualized as a network using the igraph R package [33]. Node and edge files were derived from GO term similarity analysis using NaviGO [34], with a Lin’s similarity score cutoff of 0.7.

Two sets of ‘hub-genes’ were considered for this work: 1) genes that were identified as those with gene significance and module membership > 0.8 (Fig. S5) and differential expression at one or more time points (edgeR, *p* < 0.01) (indicated in red section of Euler diagram, Figs. S1) and 2) those differentially expressed genes (*p* < 0.01 in the edgeR analysis) present in pathways that were enriched in filtrate-correlated modules (indicated in orange section of Euler diagram, Fig. S1). In total 792 genes (out of a total of 11869 transcribed genes) were identified for further analysis.

## Results

### Physiological responses of *T. pseudonana* to *C. atlanticus* filtrate treatment

To determine whether physical interaction is required for *C. atlanticus* antagonism against diatoms [10], we collected bacteria-free filtrate from mono-cultures of *C. atlanticus* cells grown without previous exposure to the diatoms. Different proportions of the bacteria-free filtrate (1.6, 3.3, 13, 33, 66, and 100 % v/v filtrate to diatom growth medium) were added to exponentially growing axenic cultures of *T. pseudonana* and compared to control cultures. Treatment with *C. atlanticus* filtrate affected the relative in vivo chlorophyll *a* fluorescence levels of *T. pseudonana* cultures in a dose-dependent manner, with a half maximal inhibitory concentration of 33% v/v (Fig. S6), indicating that part of the morphology in the diatom was induced by bacterial extracellular compounds. The increased relative in vivo chlorophyll a fluorescence at low concentrations of filtrate (<13% v/v) could reflect either an increase in in vivo fluorescence per cell, or an increase in the total number of cells; high concentrations (66, 100%) of *C. atlanticus* filtrate were deleterious to the diatom within 2 days.

**Fig. 1:**
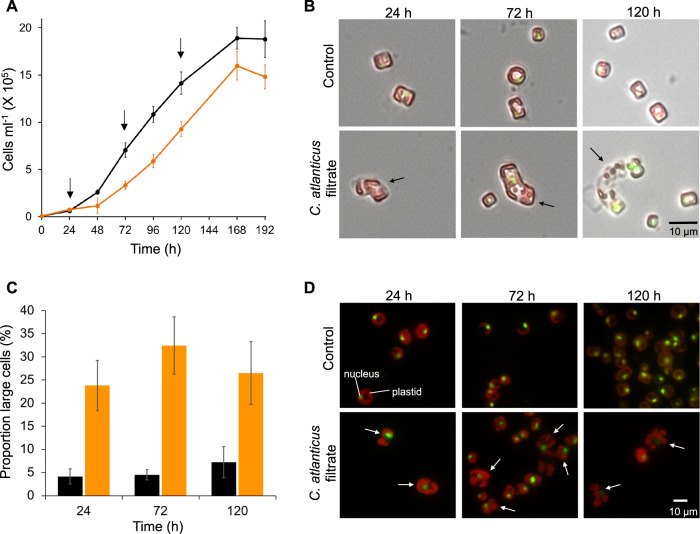
Effect of *C. atlanticus* filtrate on growth and morphology of *T. pseudonana*. **A** Growth of *T. pseudonana* cultured with 33% v/v *C. atlanticus* filtrate (orange) or with 33% v/v control bacterial media (black). Black arrows indicate sampling times for transcriptome analysis. Cell counts at 0 h were not directly measured but calculated from dilution of cultures used to initialize the experiment. **B** Representative brightfield images of *T. pseudonana* morphology grown under control conditions (top row) or in *C. atlanticus* filtrate-treated (bottom row) conditions over the course of the experiment. The three time points corresponding to transcriptome sampling time points are shown. Brightfield images show cell size and shape, overlapped with fluorescence images of SYBR Green stained DNA in diatom nuclei (green fluorescence) and chlorophyll fluorescence (red fluorescence). Black arrows indicate treated cells with abnormal morphology. **C** Percentage of control (black) and *C. atlanticus* filtrate treated (orange) *T. pseudonana* cells with a cell area 2 standard deviations larger than the mean cell area of the control population at each time point. Cell area measurements were obtained from brightfield microscopy images. **D** Representative epifluorescence images of *T. pseudonana* morphology grown under control conditions (top row) or in *C. atlanticus* filtrate-treated (bottom row) conditions over the course of the experiment. Green fluorescence represents SYBR Green stained DNA in the diatom nucleus, and red fluorescence represents chlorophyll fluorescence in plastids. White arrows indicate treated cells with abnormal morphology.

A time course of bacterial exudate impacts on diatom cell abundance, physiology, and transcriptional modulation was examined in more detail by adding 33% v/v bacterial filtrate to axenic cultures of *T. pseudonana*. The maximum cell abundance (Fig. 1A) and chlorophyll *a* fluorescence (Fig. S7A) were reduced in the filtrate-treated cultures, along with a slight decrease in specific growth rate for filtrate-treated cultures (0.77 day^−1^), as compared to control cultures (0.81 day^−1^; Fig. S7B). The photosynthetic efficiency (F_v_/F_m_) at the initial time point (24 h) was significantly lower in control samples than in filtrate-treated samples (Fig. S7C). A subsequent experiment indicated that dilution of *T. pseudonana* control cultures from 1 × 10^6^ cells/ml to 5000 cells/ml led to a comparable reduction in Fv/Fm (Fig. S7D). The filtrate-treated cells did not display a similar transient response to dilution.

Microscopy-based cell-size measurements suggested that a subset of *T. pseudonana* cells increased in size over time after exposure to *C. atlanticus* filtrate, with visible changes in cellular morphology (Fig. 1B, C, D). The larger cells contained larger chloroplasts (Fig. 1D, Fig. S8A) and an increased average nuclear area (Fig. 1D, Fig. S8B). Flow cytometry-derived forward and side scatter distributions differed between control and filtrate-treated cultures, a difference that became apparent after 12 h of exposure to the bacterial-free filtrate (Fig. S9). Forward scatter is a proxy for cell size and side scatter reflects cell shape and internal cellular structures [35]. Increases in both proxies in treated cells is consistent with the elongated phenotype observed with microscopy (Fig. 1B, C, Fig. S10). These morphological changes are similar to that observed when *T. pseudonana* was co-cultured with live *C. atlanticus* cells [10], indicating that bacterial exo-metabolites are at least partially responsible for the observed changes.

### Transcriptional responses of *T. pseudonana* to *C. atlanticus* filtrate treatment

Triplicate samples for transcriptome analysis were collected at three time points: prior to a difference in cell abundances between treatments (24 h), when the cultures were in early exponential growth phase yet cell abundances between treatments differed (72 h), and when both the control and treated cultures were in mid-late exponential growth phase (120 h, marked by arrows in Fig. 1A). The resulting transcriptomes were analyzed with two complementary methods: differential gene expression and Weighted Gene Co-expression Network Analysis (WCGNA) [31] (Fig. S1). The number of significant differentially expressed (DE) genes (*p* < 0.01, FDR < 0.05 and |log_2_ fold change | > 1) in treatment compared to control samples increased over time with 2050, 3039, and 3303 DE genes at 24 h, 72 h, and 120 h, respectively (Fig. S11A). A Mean Squared Deviation analysis of the top 500 most differentially expressed genes showed a separation of samples based on treatment and, to a lesser extent, sampling time, with replicate samples grouping together (Fig. S11B). Differentially expressed genes were enriched in ribosome biogenesis, RNA translation, amino acid metabolism, response to stress, RNA modification, and transport at all three timepoints (Fisher’s exact test; Table S1).

**Fig. 2:**
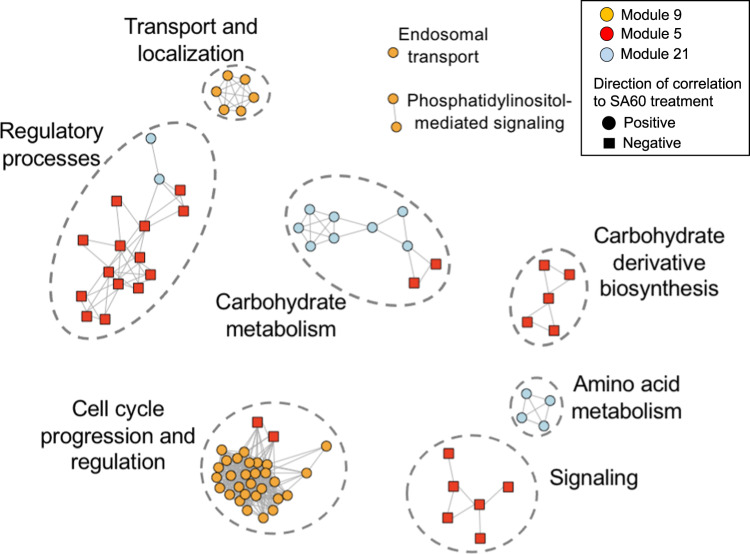
Functional enrichment of genes belonging to *C. atlanticus* filtrate treatment related modules. Network of enriched GO terms in modules 9, 5, and 21 based on a Lin’s similarity score cutoff of 0.7. Enrichment analysis was done on each module separately. Each node represents an enriched GO term while clusters represent GO terms of similar function. Enriched terms are colored by the module they belong to and shaped based on directionality of correlation to *C. atlanticus* filtrate treatment. The length of edges connecting the nodes is arbitrary.

We used WCGNA to cluster *T. pseudonana* genes into modules of genes with comparable transcription profiles. The resulting 12 modules were correlated (Pearson) with measured experimental parameters and phenotypic changes observed in the diatom with or without bacterial filtrate treatment (Table S2). Most modules were correlated with either i) bacterial filtrate treatment-dependent traits (percent of enlarged cells, maximum cell diameter, and minimum cell diameter; modules 9, 5 and 21), or ii) time-dependent traits (chlorophyll *a* fluorescence, cell count, and sampling time; modules 11, 1, 35 and 34) (Fig. S4). Modules correlated with bacterial filtrate-treatment (9, 5, 21) were enriched in cell cycle progression, carbohydrate metabolism and biosynthesis, amino acid metabolism, transcriptional regulation and signaling genes (Fig. 2; Table S1). In contrast, modules correlated with time-dependent traits were enriched (*p* < 0.01) in transport, localization, RNA processing, ribosomal biogenesis, catabolic processes, and transcriptional regulation (GO Biological Process terms; Table S1). These functional features were also identified by differential expression analysis, highlighting the importance of separating time-dependent and treatment-dependent traits.

**Fig. 3:**
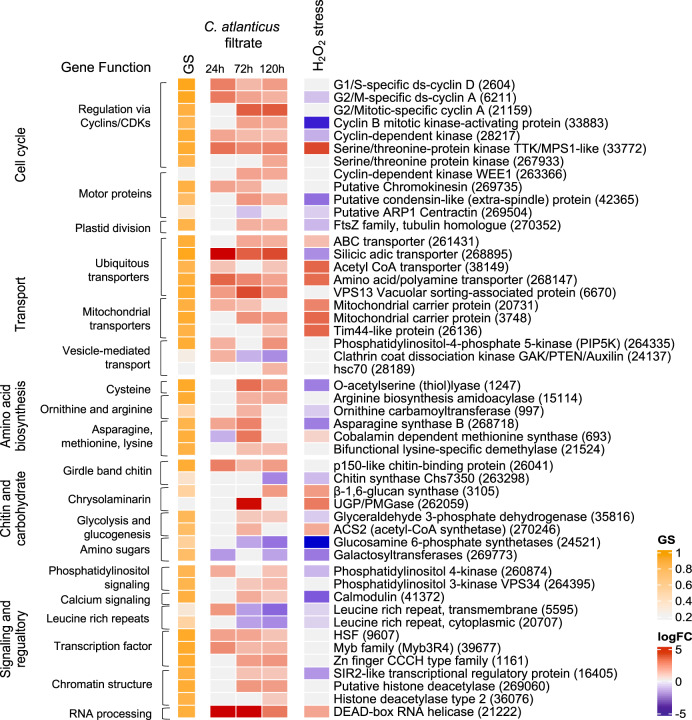
Transcriptional patterns of selected *T. pseudonana* genes in response to *C. atlanticus* filtrate treatment and exposure to oxidative stress. Representative genes with functions in cell cycle progression, transport, amino acid biosynthesis, chitin and carbohydrate metabolism, and signaling and regulatory processes are shown. Columns from left to right: gene significance (GS) for *C. atlanticus* filtrate, differential expression (logFC) of genes in response to *C. atlanticus* filtrate treatment at 24, 72, and 120 h after exposure as compared to control cultures, and differential expression of *T. pseudonana* after 2 h of H_2_O_2_-induced oxidative stress compared to control conditions (see Methods). Labels on the right indicate encoded protein, with gene ID in parentheses.

Further analysis of genes that were both differentially expressed and correlated to the *C. atlanticus* filtrate treatment (Fig. S1) highlighted five cellular pathways in which expression patterns were significantly altered by *C. atlanticus* filtrate treatment: cell cycle progression; transport; amino acid metabolism; chitin and carbohydrate metabolism; and recognition, signaling and regulation (Fig. 3). The *C. atlanticus* filtrate induced changes in the abundance of transcripts associated with numerous components of cell cycle regulation: kinase complexes, including regulatory cyclin subunits known to control progression through G1/S, G2/M and mitosis stages of the cell cycle; cyclin dependent kinases (CDKs) that act as catalytic subunits (Fig. 3, Table S3); putative casein kinases that inhibit CDK activity; [36] and the mitotic spindle checkpoint protein MAD-2 (Table S3). In addition to regulatory genes, several hub genes (see methods) encoded motor proteins involved in chromosome segregation, kinetochore binding and spindle formation, including a chromokinesin, centrosome-associated actins, and condensin-like proteins (Fig. 3, Table S3). Transcripts encoding the plastid division protein FtsZ are elevated at 72 h and 120 h in the treated cultures as compared to control cultures, coinciding with the increased chloroplast content in treated diatom cells at these timepoints (Fig. 3, Fig. S8A).

Filtrate treatment resulted in upregulation of transcription of genes involved in carbon metabolism such as the TCA cycle, glycolysis and glucogenesis, and chrysolaminarin-based storage mechanisms (Fig. 3, Table S3). We also observed an increase in transcripts associated with amino acid biosynthesis pathways in treated cells, with changes associated with biosynthesis pathways of cysteine; aspartate and alanine; valine, leucine and isoleucine; asparagine, threonine, methionine and lysine; and ornithine and arginine (Fig. 3, Table S3). Silicic acid transporter-encoding transcript abundance (Sit1 and Sit3) was also upregulated (Fig. 3, Table S3). In contrast, the transcript abundance decreased for twenty genes that encode chitinases and chitin synthases and the enzymes required for biosynthesis of cell-wall-associated chitin polymers (Table S3). The treatment also resulted in downregulation of genes that encode proteins localized to the girdle band region of the cell wall that regulate the stability of the cell wall during cell division, which suggests a destabilization and increased permeability of the cell wall of cells treated with *C. atlanticus* filtrate (Fig. 3, Table S3).

To understand whether the observed transcriptional responses were specific to the bacterial exudate or represented a general cellular response to stress, we evaluated the stress response of *T. pseudonana* to H_2_O_2_-induced oxidative stress after 2 h (Fig. 3). A large number (1569) of genes were differentially expressed under both H_2_O_2_-induced oxidative stress and *C. atlanticus* filtrate treatment and included genes involved in cell cycle regulation, signaling, and amino acid metabolism (Fig. 3, Fig. S12, Table S1). Genes enriched in processes related to stress response, amino acid biosynthesis, photosynthesis, and general metabolism were differentially expressed in oxidative stress or in both oxidative stress and *C. atlanticus* filtrate treatments. Conversely, genes differentially expressed specifically in response to *C. atlanticus* filtrate treatment were enriched in cell cycle control, including DNA replication and microtubule-based movement (Table S1, Fig. 3). Although oxidative stress and ROS signaling can play an important role in microbial interactions [37–39], we did not find transcriptional evidence that *C. atlanticus* filtrate induced an oxidative stress response in *T. pseudonana*. Together these results indicate that observed changes in transcription of genes involved in cell cycle control are due to the filtrate treatment rather than a general response to stress.

### Phenotypic assessment of transcriptome-identified pathways

**Fig. 4:**
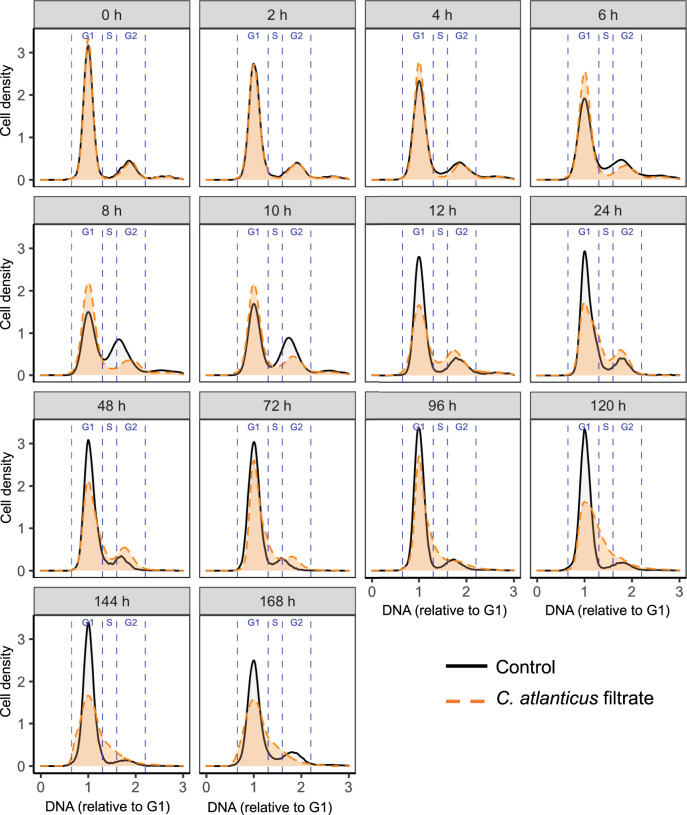
Cell cycle analysis via flow cytometry in control (black) and *C. atlanticus* filtrate treated *T. pseudonana* cells (orange). DNA distributions of representative cytograms are shown (one representative replicate out of three is shown here), with distributions normalized to 1 μm beads and aligned based on the mode of G1 peaks. Each plot represents samples taken at indicated times after initial treatment with the filtrate. Blue dashed lines indicate boundaries of different cell cycle stages (G1, S, G2) and were drawn based on the signal of the control cultures.

The *C. atlanticus* filtrate induced changes in the abundance of transcripts associated with cell cycle regulation. We confirmed disruption of cell cycle progression in treated *T. pseudonana* cells using flow cytometry-based cell cycle analysis (Fig. 4, Fig. S13). Under control conditions, the proportion of control cells in S phase decreased and the proportion of cells in G1 phase increased between 6–12 h after the start of the experiment, indicating active cell division (Fig. 4). In contrast, in treated cultures the proportion of S phase versus G1 phase remained constant during this same interval, suggesting an almost immediate disruption of cell cycle progression in the treated cultures. The difference in DNA distributions between control and treated cultures became more apparent over time with a gradual increase in the proportion of treated cells in S and G2 phases after 96 h. During late exponential and stationary phases of growth (120–168 h), a large proportion of cells in the treated cultures were arrested in S phase and a subset of treated cells displayed DNA content greater than G2, reflecting unregulated DNA replication and the presence of larger nuclei, in accordance with the differential expression patterns of cell-cycle related genes (Figs. 3, 4).

**Fig. 5:**
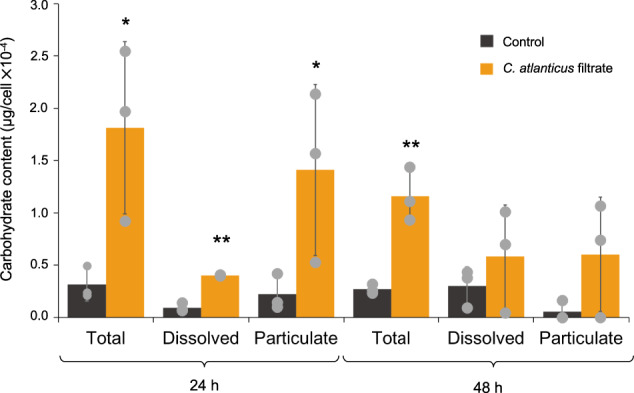
Carbohydrate content in control (black) and *C. atlanticus* filtrate treated (orange) diatom cultures. Gray dots represent individual experimental replicates, stars indicate significant difference between control and filtrate treated cultures at each time point by students *t*-test (*p < 0.05, **p < 0.01). Total, dissolved, and particulate carbohydrate measurements were derived from bulk volume measurements normalized to cell concentration to get a carbohydrate content per cell. Particulate carbohydrate content was calculated as the difference between total and dissolved fractions. In filtrate treated samples, carbohydrate content of pure bacterial filtrate was subtracted from sample measurements to estimate carbohydrates produced by diatoms.

Treatment with *C. atlanticus* filtrate also resulted in changes in transcript levels associated with amino acid and carbohydrate metabolism as well as cell wall components (Fig. 3). These transcriptional profiles suggest an upregulation of these biosynthesis pathways that, with a coinciding disruption of the cell wall stability, could potentially result in enhanced release of organic compounds. To test whether diatoms exposed to *C. atlanticus* filtrate produce and exude higher levels of complex carbohydrates, we subjected cultures to 24 h and 48 h of *C. atlanticus* filtrate and analyzed the extracellular, total, and particulate carbohydrate content of these treated diatoms relative to control cultures. Diatoms were treated with undiluted filtrate to ensure a rapid and uniform response by treated cells (Fig. S14). We observed an increase in extracellular carbohydrates after both 24 h and 48 h of treatment in total carbohydrate content, indicative of increased carbohydrate production (Fig. 5). The increase in the dissolved phase suggests an increased release of carbohydrates by the diatom into the media (Fig. 5), in accordance with the transcriptomic results.

## Discussion

Interactions between diatoms and bacteria are mediated through a series of metabolic exchanges that can influence the behavior and metabolism of both organisms. We focused here on a previously characterized antagonistic interaction between the model diatom *T. pseudonana* and the bacterium *C. atlanticus* [10]. In co-culture, live bacteria attach to the surface of diatom cells, inhibit cell division, and modify the morphology of the diatom [10]. Here, we developed an experimental system that relies on bacterial exudates rather than the presence of live bacteria. We found that the *T. pseudonana* morphology of increased cell size with larger chloroplast area and nuclei arises during exposure to bacterial exudates without the presence of live bacteria. In addition, the observed increase in production of carbohydrate-based compounds and inferred increase in amino acid production and cell wall permeability suggested that released bacterial metabolites may modify the metabolism of the diatom to better support bacterial growth. Resulting changes in the diatom’s morphology, especially the increased cell size, could have far reaching ecological consequences, including increased sinking rates and grazing susceptibility, and affect light absorption patterns and export of carbon from the surface ocean [40–42].

**Fig. 6:**
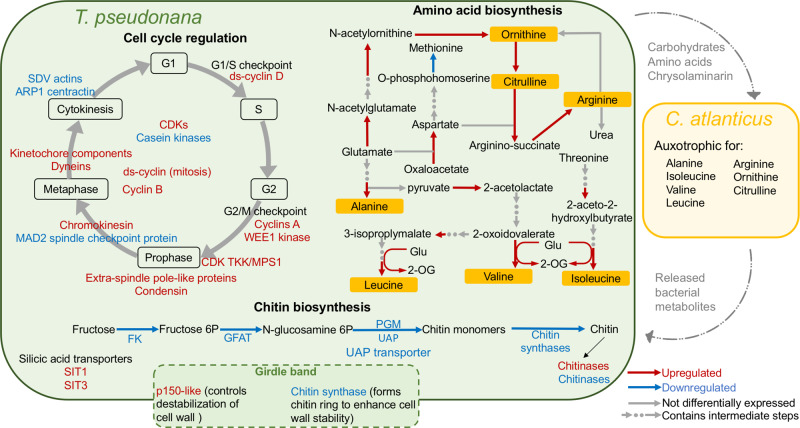
Summary of *T. pseudonana* transcriptional changes in response to *C. atlanticus* filtrate. The green box represents *T. pseudonana*, the yellow box represents *C. atlanticus*. Upregulated (red) and downregulated (blue) *T. pseudonana* genes involved in cell cycle progression, amino acid metabolism, and chitin biosynthesis are shown. Colored letters and arrows represent up or downregulated genes, grey arrows represent genes that are not differentially expressed. Arrows with dots indicate intermediate steps not shown in the pathway. *C. atlanticus* auxotrophic amino acid residues are derived from van Tol et al., 2017 [10], and potential interacting metabolites are indicated with the curved grey arrows.

A schematic based on our transcriptome and phenotypic analyses summarizes how extracellular metabolites of *C. atlanticus* impact *T. pseudonana* physiology (Fig. 6). Transcriptional changes in diatom cell cycle progression, silicic acid uptake, and chitin metabolism corresponded to the observed morphology. The combination of flow-cytometry-based cell cycle analysis with the larger nuclei observed in microscopy and the differentially expressed genes in the treated cells indicate that released *C. atlanticus* metabolites inhibit *T. pseudonana* cell cycle progression. Changes in cell cycle progression were evidenced by disruption of genes encoding numerous regulatory and motor proteins involved in division (Fig. 6), as well as a gradual increase in the proportion of S and G2 phase cells (Fig. 4). The atypical increase in S phase cells with *C. atlanticus* filtrate treatment (Fig. 4) could indicate replication stress, which can lead to uneven chromosome segregation and aneuploidy in eukaryotic cells [43]. We observed transcriptional changes in genes responsible for spindle formation and chromosome segregation (Fig. 6); analogous genes in the centrosome of other eukaryotic cells were associated with mitotic delay and genomic instability [44]. Similarly, observed transcriptional changes in kinetochore function (Fig. 6) have been linked to development of aneuploidy, as kinetochores are responsible for binding microtubules, coordinating chromosome movements, and activating spindle checkpoints [45]. Additionally, the downregulation of centrosome-associated actins involved in spindle separation and contractile ring formation during cytokinesis suggests that the filtrate inhibits cytokinesis of *T. pseudonana* cells [46], leading to enlarged diatom cells that continue to replicate their DNA but are unable to divide (Fig. 1, Fig. 6). Previous work shows that bacterial quorum sensing molecules cause cell cycle arrest in coccolithophores, while at the same time protecting affected cells from viral mortality, highlighting the importance of bacterial signals in multitrophic interactions [47]. The multitrophic effect of cell cycle changes in *T. pseudonana* warrants further investigation and could have implications for viral or grazing susceptibility. Cell volume in *Thalassiosira* is constrained by G1 DNA content [48]; as long as cell metabolism is active in aneuploid or polyploid cells unable to divide, an increase in size is expected. Although bacterial algicides can induce cell cycle arrest and programmed cell death [49], live diatom cells displaying the morphology induced by the *C. atlanticus* filtrate continued to photosynthesize (Fig. S7A) and produce organic material bioavailable to bacteria (Fig. 5).

Enlargement of the silica cell wall is a necessary accompaniment for cell expansion; we observed an upregulation of genes encoding silicic acid transporters suggesting that *T. pseudonana* increased silicic acid uptake (Fig. 3) [50]. Similarly, transcriptional patterns of genes encoding girdle band localized proteins involved in chitin synthesis and cell wall stability suggested a destabilization of the silica cell wall, which could also allow for the observed cell enlargement (Fig. 6). The predominant downregulation of genes required for chitin synthesis suggested a decoupling of silicon precipitation and chitin utilization, potentially leading to an underproduction of extracellular chitin fibers that slow the sinking rate of diatoms. Both filtrate-treated *T. pseudonana* cells and *T. pseudonana* co-cultured with live *C. atlanticus* sank to the bottom of the tube [10].

Bacterially produced compounds may manipulate the metabolism of the diatom to support bacterial growth. This can be achieved by specific enhancement of biosynthesis of compounds beneficial for the bacteria and/or enhanced export of metabolites from the diatom to the phycosphere. Repression of cell division may also indirectly support bacterial growth and allow for an extended leakage of metabolites as cells continue to photosynthesize and fix carbon without dividing. Such mechanisms have been observed in plants; polyploidy is common in cells near sustained nutrient exchange sites between the host and biotroph, where enlarged host cells can support the growth of their biotroph by producing excess carbon [51]. We found that treated *T. pseudonana* cells released twice as many dissolved carbohydrates compared to untreated cells (Fig. 5), along with an upregulation of genes involved in chrysolaminarin synthesis, glucogenesis, and glycolysis (Figs. 3, 6). *C. atlanticus* has the genetic potential to utilize these substrates as a nutrient source using the numerous carbohydrate-active enzymes (CAZymes) encoded in its genome [10]. We also observed an upregulation of multiple genes required for the biosynthesis of alanine, valine, leucine, isoleucine, and arginine, amino acids in *T. pseudonana* for which *C. atlanticus* is auxotrophic [10, 52]. Previous studies showed that the bacterium is able to use external amino acids as a sole nitrogen source [10, 52]. In plant rhizospheres, where the host provides a stable availability of select metabolites, bacterial auxotrophy for specific amino acids can provide a selective fitness advantage by allowing them to conserve the energy required to synthesize these essential compounds [53]. The postulated destabilization of the silica cell wall and chitin structures could lead to an increased permeability of the cell wall, potentially exacerbating the leakage of metabolites from *T. pseudonana* that can support bacterial growth. Therefore, by producing compounds that increase diatom leakiness and production of carbohydrates and essential amino acids, *C. atlanticus* may create a beneficial nutrient rich micro-environment. Leakage-facilitated metabolic exchanges among phytoplankton were described in the Black Queen Hypothesis, where one microorganism relies on a neighbor to produce essential components and thereby conserves its own metabolic potential [54, 55].

Diatoms can select and modulate the composition of their microbiome through the excretion of metabolites [18–20]. The antagonistic bacterium described here may be maintained within a diatom microbiome due to the heterogeneity of the diatom’s response at the individual cell level. In our experiments in which *T. pseudonana* cultures are exposed to 33% bacterial filtrate, both flow cytometry and microscopy observations indicate that some of the cells exhibit aberrant morphology while other cells appear healthy. This morphological heterogeneity can be the result of different metabolic states of the cells, difference in the exact amount of bacterial metabolites each cell absorbed, or a random ‘bet hedging’ strategy [56–59]. Additionally, antagonistic bacteria can be maintained with the diatom through regulation by other bacteria [60–62]. *C. atlanticus* was originally isolated from a consortium of bacteria from the diatom *P. multiseries*, and co-culture of live bacteria induced growth inhibition on a wide range of phylogenetically diverse axenic diatoms [10]. Our bacteria-free system mimicked the overall physiological response of *T. pseudonana* co-cultured with live *C. atlanticus*. *T. pseudonana* response to the bacterial filtrate is dose-dependent, with high concentrations being toxic and low concentrations stimulating fluorescence. The increased relative in vivo chlorophyll a fluorescence at low concentrations of filtrate (< 13% v/v) suggests either an increase in in vivo fluorescence per cell or an increase in the final concentration of cells, perhaps due to hormesis, an apparent increase of viability likely due to enhanced reparative processes due to stress [63]. In contrast to the direct association with live bacteria, we did not observe significant induction of multiple nuclei within individual cells. These differences between the diatom response to live bacterial co-culture and bacteria-free exudates can be a result of several factors, for example, bacterial attachment or close proximity to the diatom can increase exudate concentrations in the diatom phycosphere. Additionally, a yet unexplored diatom-bacteria metabolic crosstalk may alter the pathogenicity of the bacteria and its exudates.

The interactions between phytoplankton and bacteria are dynamic and can change from beneficial to algicidal based on chemical cues released by the host [64]. Although active metabolites released by *C. atlanticus* were produced in the absence of previous exposure to the diatom, the bacteria were grown on a rich media, which may have simulated a nutrient-rich phycosphere environment. Nonetheless, our study indicates that diatom-specific cues are not required for the production of algicidal compounds by *C. atlanticus*. Future work warrants further investigation of how the presence of phytoplankton and phytoplankton-produced compounds impacts the production of algicides by *C. atlanticus*.

Diverse bacterially produced compounds ranging from small molecules to enzymes have algicidal impacts on phytoplankton. For example, bacterial quorum-sensing molecules can inhibit diatom growth and metabolism [24, 25, 47]. Similarly, a flavobacterium produces L-amino acid oxidases, exuding hydrogen peroxide as a byproduct of amino acid oxidation, resulting in an indirect algicidal effect on green algae [64]. The active components of released *C. atlanticus* metabolites are currently unknown, however, the *C. atlanticus* genome encodes cyclomodulins such as gamma-glutamyl transpeptidases that are known to affect the cell cycle of various eukaryotic cells [65, 66]. This study opens the way for further investigations into the isolation and characteristics of bioactive components produced by a ubiquitous marine bacterium. Flavobacteria are known to play important roles in organic matter degradation during phytoplankton blooms [67] and elucidating the effects of bacterially produced metabolites on diatoms has important implications for understanding bloom dynamics and nutrient flux in the ocean.

## Supplementary information

Supplementary information

Supplementary Table 1

Supplementary Table 2

Supplementary Table 3

## Data Availability

All sequencing data is publicly available on NCBI under the accession number GSE197934. Code used for data processing and statistical analysis is available at GitHub (https://github.com/armbrustlab/Thaps_Catl). All supplementary data tables and secondary data is available on Zenodo (10.5281/zenodo.6672614). The *C. atlanticus* SA60 strain used in these experiments is available at Bigelow National Center for Marine Algae and Microbiota under the identifier NCMA B36.
