# Supplementary material for: Flavobacterial exudates disrupt cell cycle progression and metabolism of the diatom Thalassiosira pseudonana
Source: ISME J. 2022 Sep 14;16(12):2741–51. doi: 10.1038/s41396-022-01313-9 (PMC9666458; doi:10.1038/s41396-022-01313-9)
Supplement: Supplementary file 1 — Supplementary information [file 41396_2022_1313_MOESM1_ESM.pdf]

Supplementary Information for

Flavobacterial exudates disrupt cell cycle progression and metabolism of the diatom *Thalassiosira pseudonana*

Zinka Bartolek[1], Shiri Graff van Creveld[1], Sacha Coesel[1], Kelsy Cain[1], Megan Schatz[1], Rhonda Morales[1], E. Virginia Armbrust[1*]

[1]School of Oceanography, University of Washington, Seattle, WA 98195

*Corresponding author:

E. Virginia Armbrust

University of Washington

Seattle, WA 98195

Phone: 206-616-1783

Email: armbrust@uw.edu

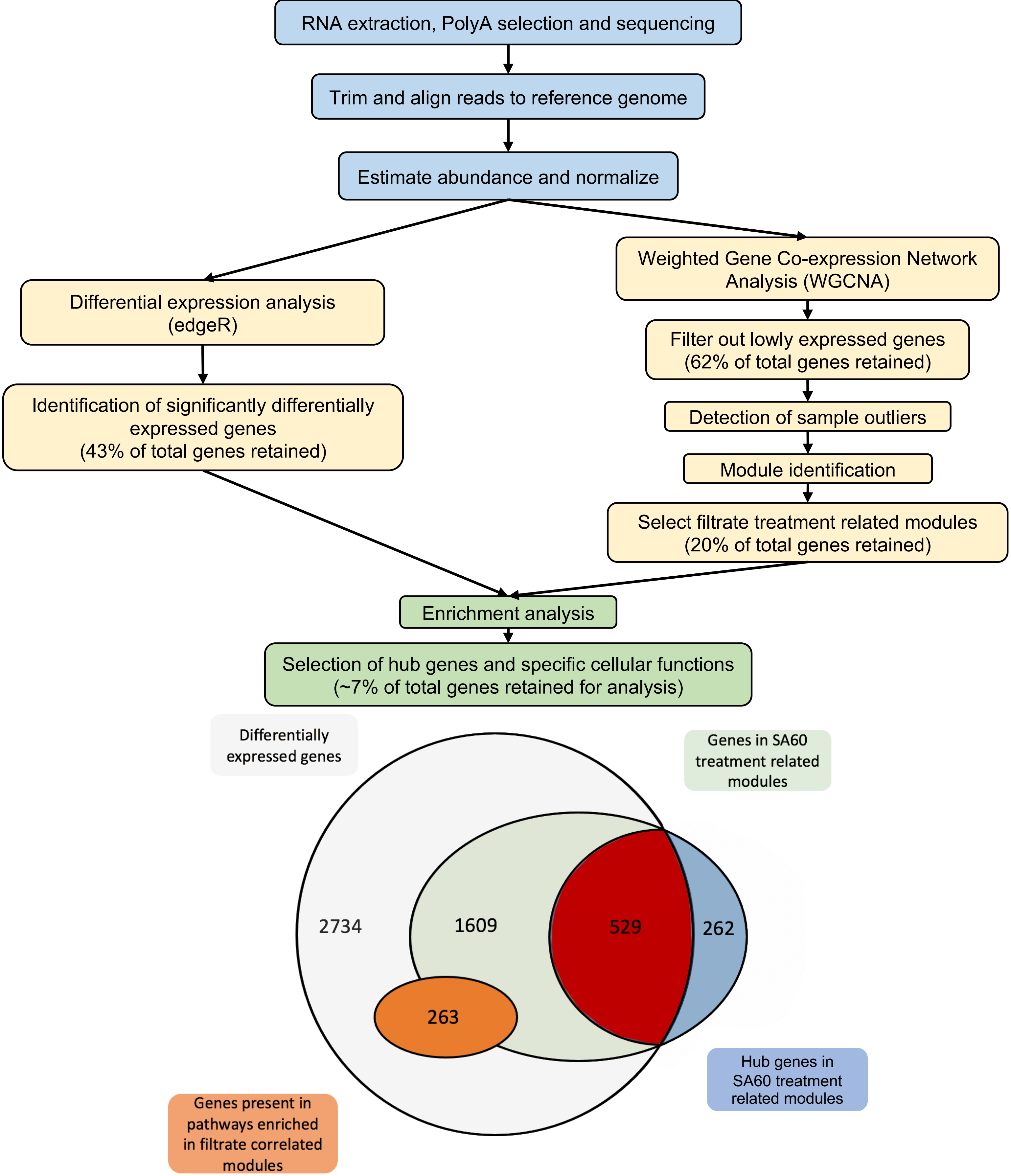

Figure S1. Flowchart of statistical approach used for analyzing transcriptome data. Sample pre-processing (blue boxes), statistical analysis using WGNA clustering and differential expression analysis (yellow boxes), and selection of interesting genes (green boxes) is shown. Euler diagram illustrates the selection of genes considered for detailed analysis. An intersection of genes that were significantly differentially expressed in the *C. atlanticus* filtrate treated samples compared to the control in one or more of the time points sampled and hub genes identified from the four WCGNA modules were considered (red), along with a subset of genes present in pathways that were enriched in filtrate correlated WGCNA modules (orange). In total 792 genes were considered in detail for further analysis, indicated by the red and orange sections.

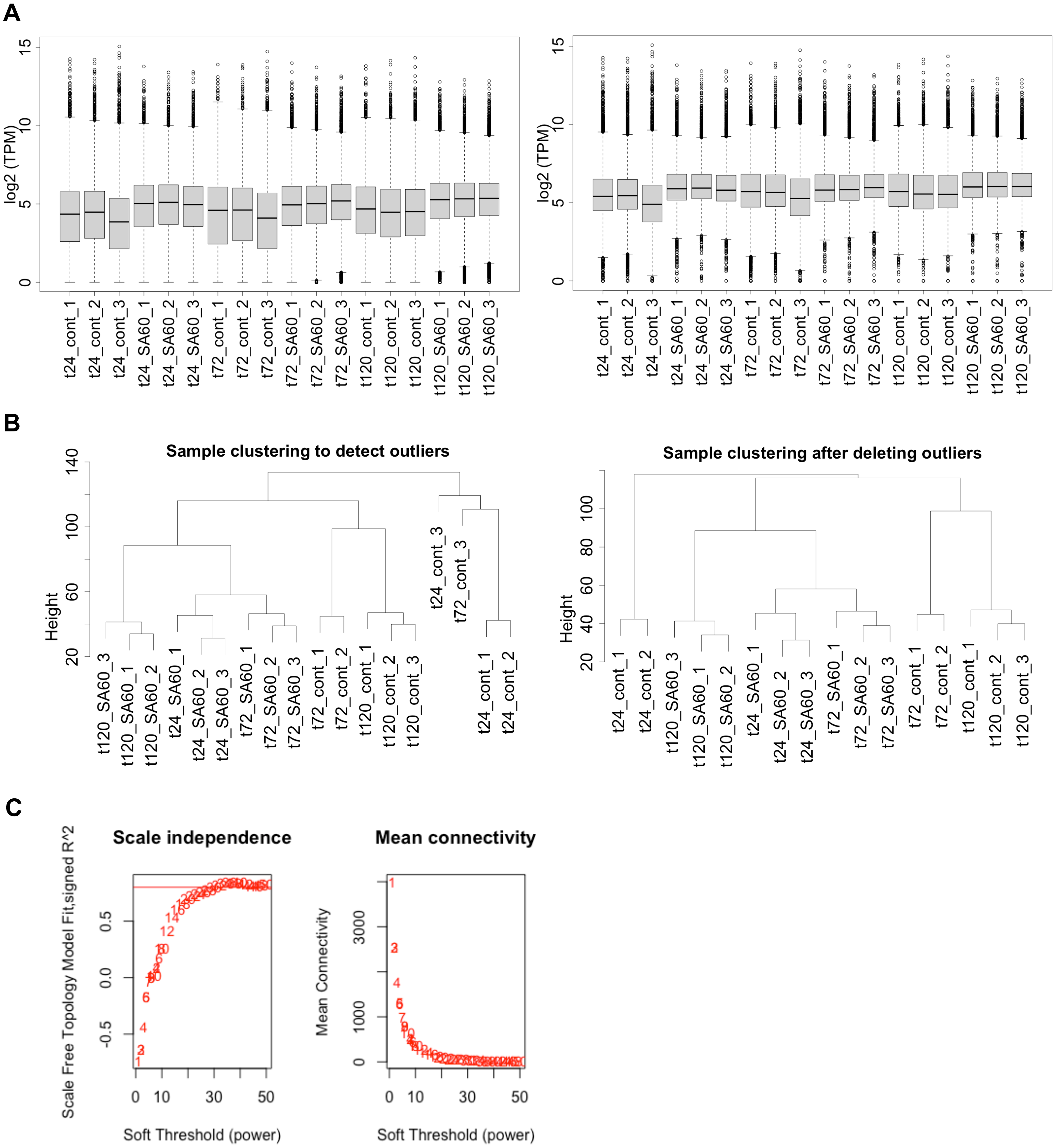

Figure S2. WGCNA sample pre-processing and module construction metrics. **(A)** Bar plots of log_2 TPM distributions in each of the collected samples before pre-processing (left) and after removing genes with expression levels below the median TPM in greater than 25% of the samples (right). Median TPM was calculated across all genes and samples. **(B)** Sample clustering dendrograms before (left) and after (right) removal of two outlier samples. **(C)** Scale-free topology and mean connectivity parameters used in network construction. A soft threshold power of 18 was used for network construction. The red line in the left panel represents the 0.8 cutoff value of the scale-free topology.

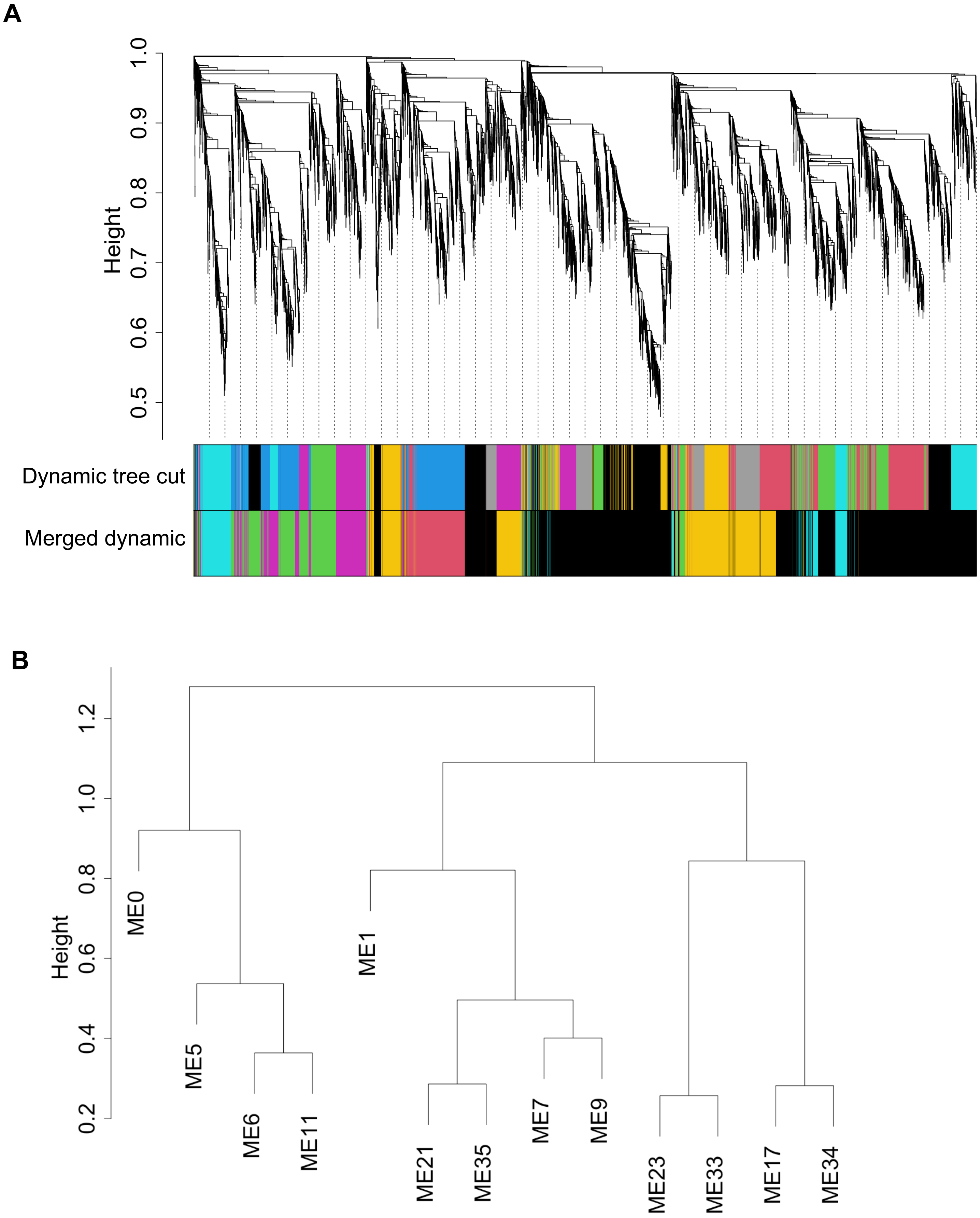

Figure S3. W**GCNA network and module construction. (A)** Dendrogram of all genes clustered via hierarchical clustering according to a dissimilarity measure (1-TOM), and the corresponding module colors. The top row of module colors indicates module assignments using a dynamic tree cut method, while the bottom row indicates module assignments after similar modules have been merged based on a module dissimilarity threshold height of 0.25. **(B)** One-dimensional clustering of merged modules according to module eigengenes, which correspond to the principal component of each module. The ME0 module captures genes that do not show strong clustering with genes in any other module.

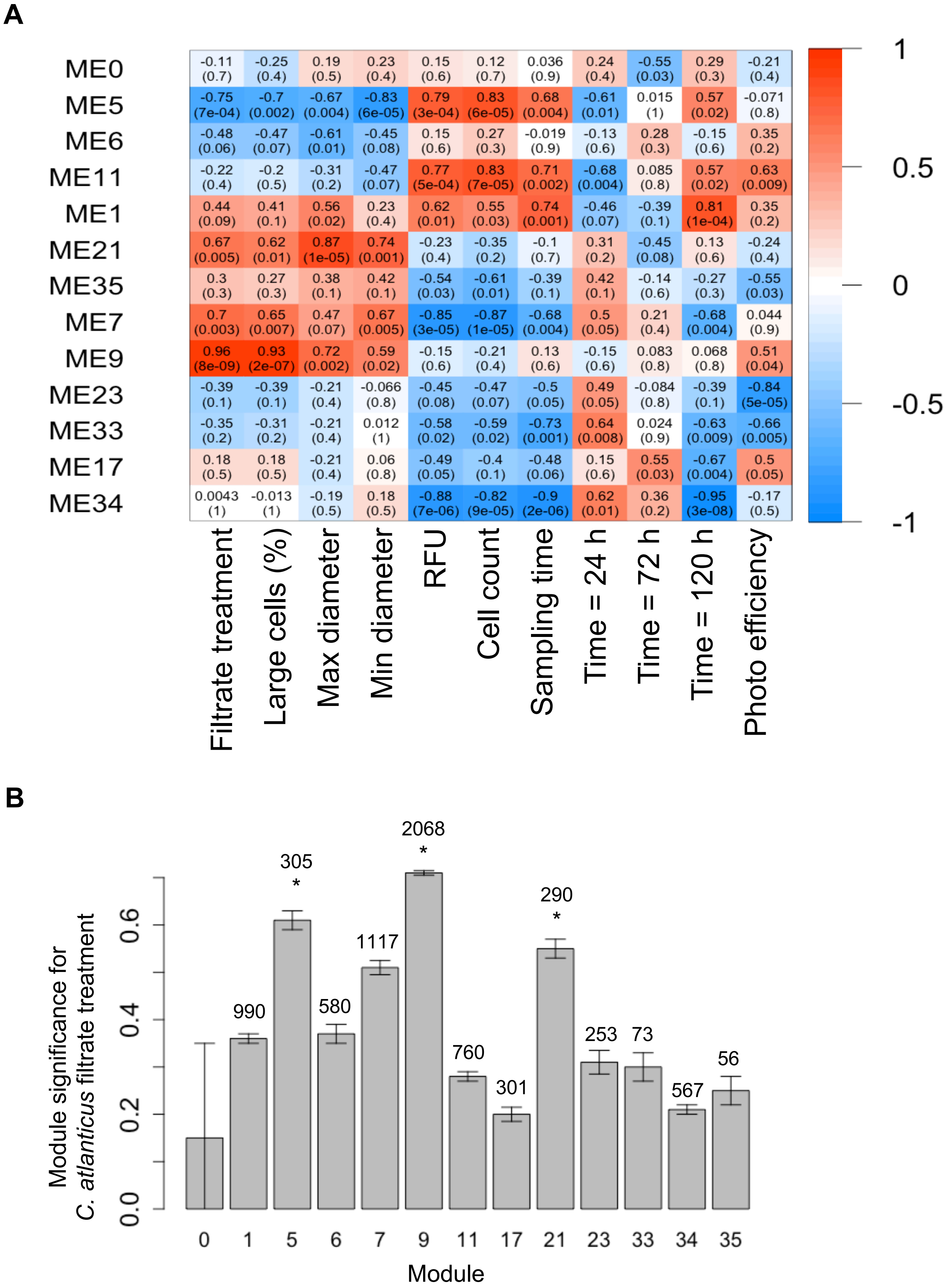

Figure S4. Correlation of WGCNA generated gene modules to *C. atlanticus* filtrate treatment and other experimental traits. (A) Heatmap of the correlation (Pearson) between the experimental traits (columns) and WGCNA-derived module eigengenes (MEs) (rows). Within each cell, upper number is the correlations of the corresponding MEs and traits; *p* values from the linear mixed-effects model in parentheses. Red indicates positive correlations, blue indicates negative correlations, according to the color legend. RFU indicates relative fluorescence units corresponding to Chlorophyll *a* content. Cell size parameters are from microscopy analysis. **(B)** Mean gene significance of each module to treatment with *C. atlanticus* filtrate. Error bars represent standard deviation across all genes in a module. Stars indicate modules with greatest significance to treatment; numbers indicate number of genes in each module.

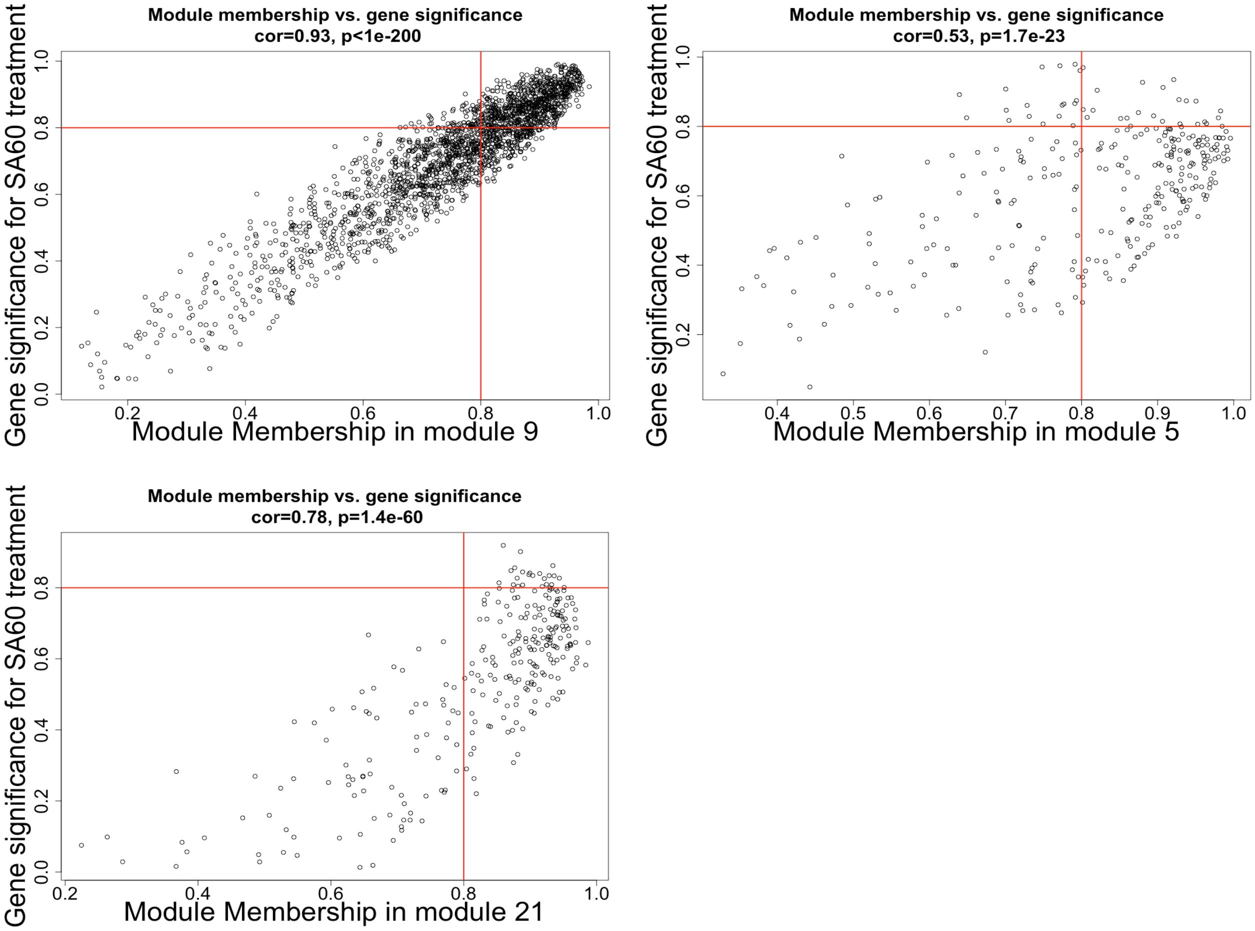

Figure S5. Selection of hub genes that are differentially expressed and correlated to the *C. atlanticus* filtrate treatment. Correlation between gene significance for *C. atlanticus* treatment traits and module membership in module 9, 5 and 21. A hub gene is defined as a gene in one of these modules with both a gene significance > 0.8 and a module membership > 0.8 (red lines).

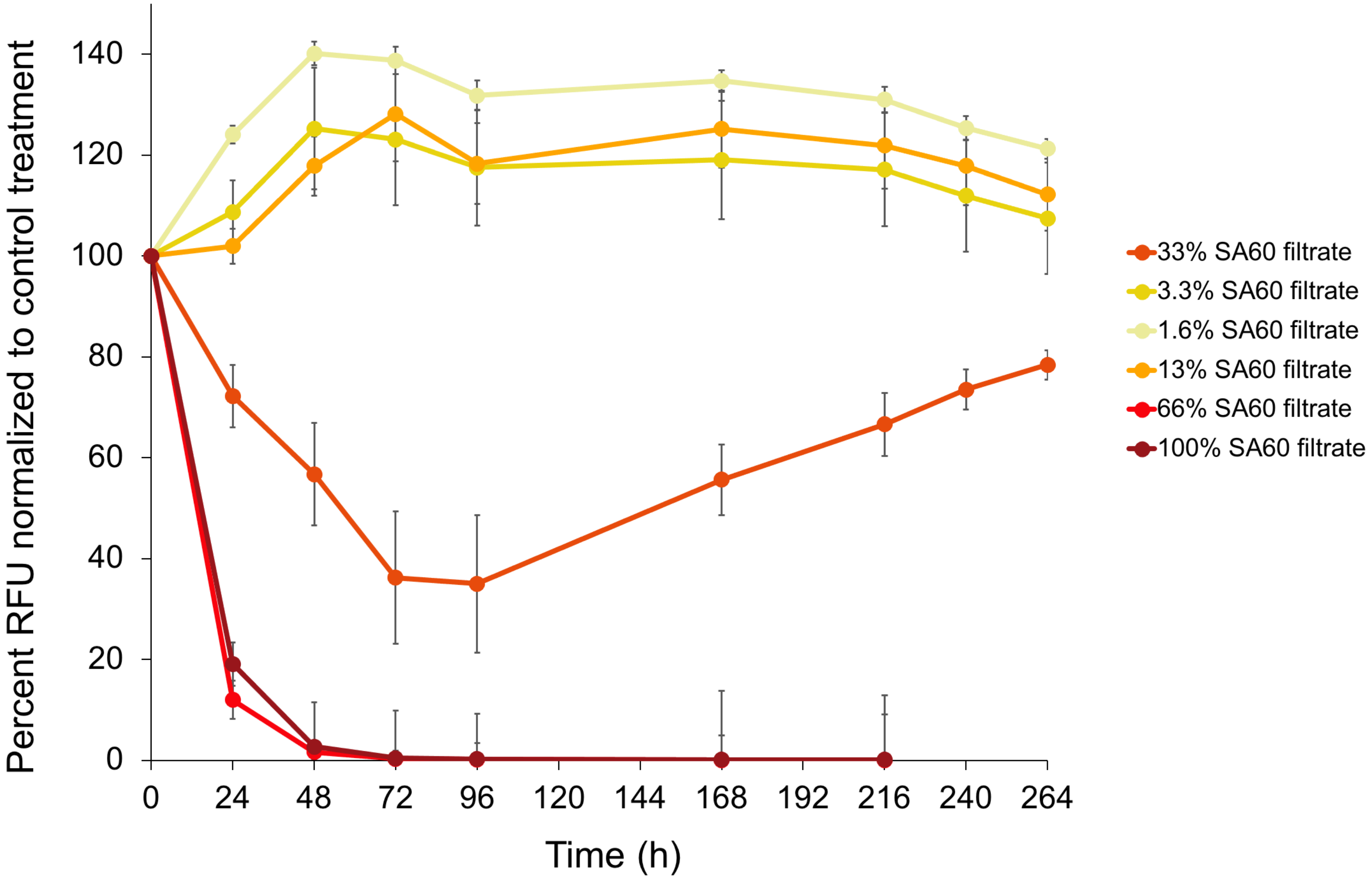

Figure S6. Dose-dependent relative fluorescence ratios of *T. pseudonana* in response to *C. atlanticus* filtrates. Relative chlorophyll fluorescence units (RFU) in response to different concentrations of *C. atlanticus* filtrate. Fluorescence under varying concentrations of *C. atlanticus* filtrate is normalized to fluorescence under control conditions. The mean of three replicate cultures is shown, error bars represent standard deviations.

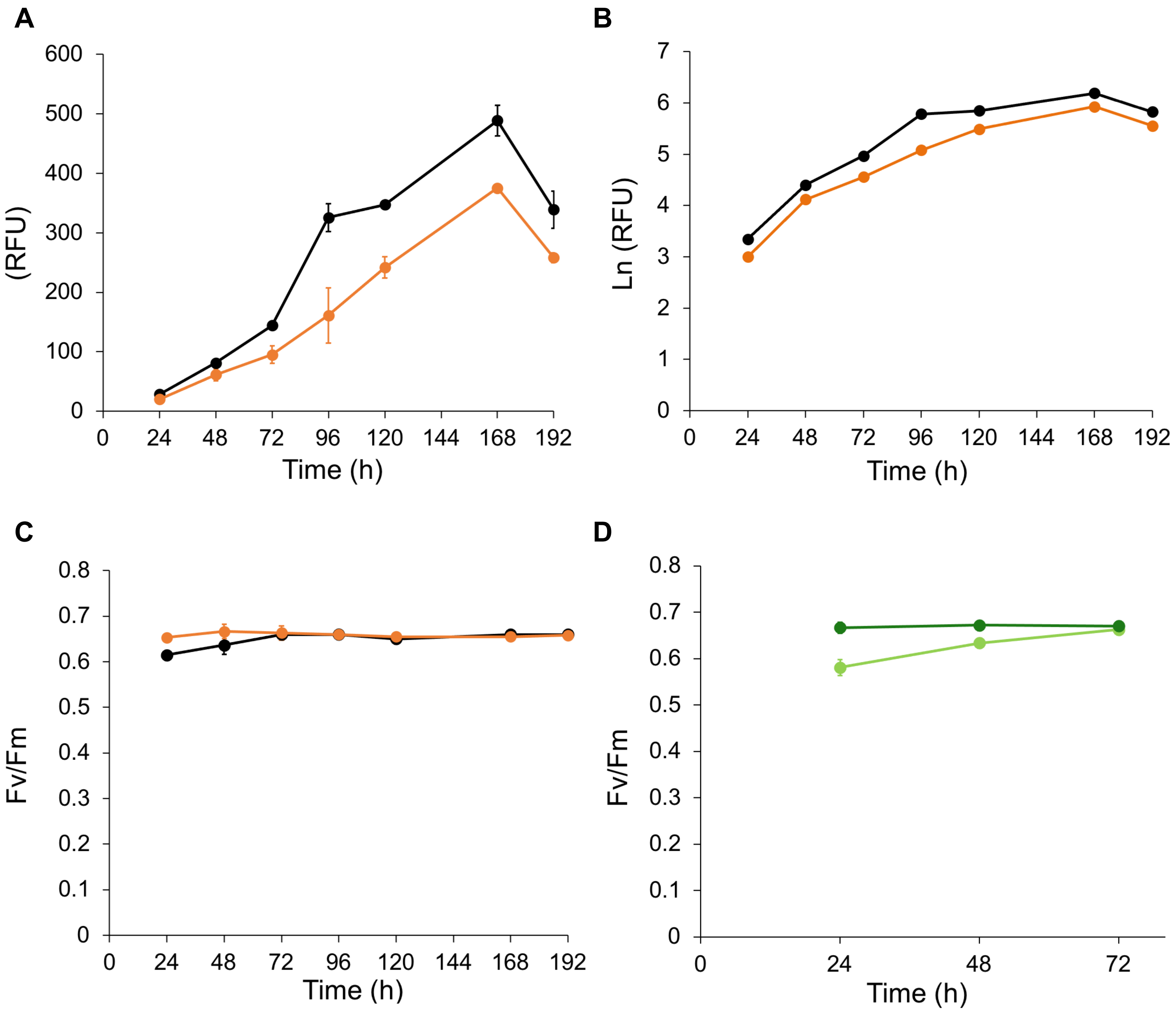

Figure S7. Additional measurements of control (black) and filtrate treated (orange) diatom cultures (A) Relative chlorophyll *a* fluorescence of *T. pseudonana* in response to 33% v/v *C. atlanticus* filtrate measured in relative fluorescence units (RFU). **(B)** RFU plotted on a logarithmic scale, with specific growth rates calculated between 24 h and 96 h of diatom growth. **(C)** Photosynthetic efficiency (F_v/F_m) of control samples and samples treated with 33% v/v *C. atlanticus* filtrate over the growth curve. (**D**) Fv/Fm of only control cultures measured during an independent experiment where control cultures were diluted to 5000 cells/ml (light green) compared to $5x10^4$ cells/ml (dark green). Error bars represent the standard deviation of triplicate cultures in all panels.

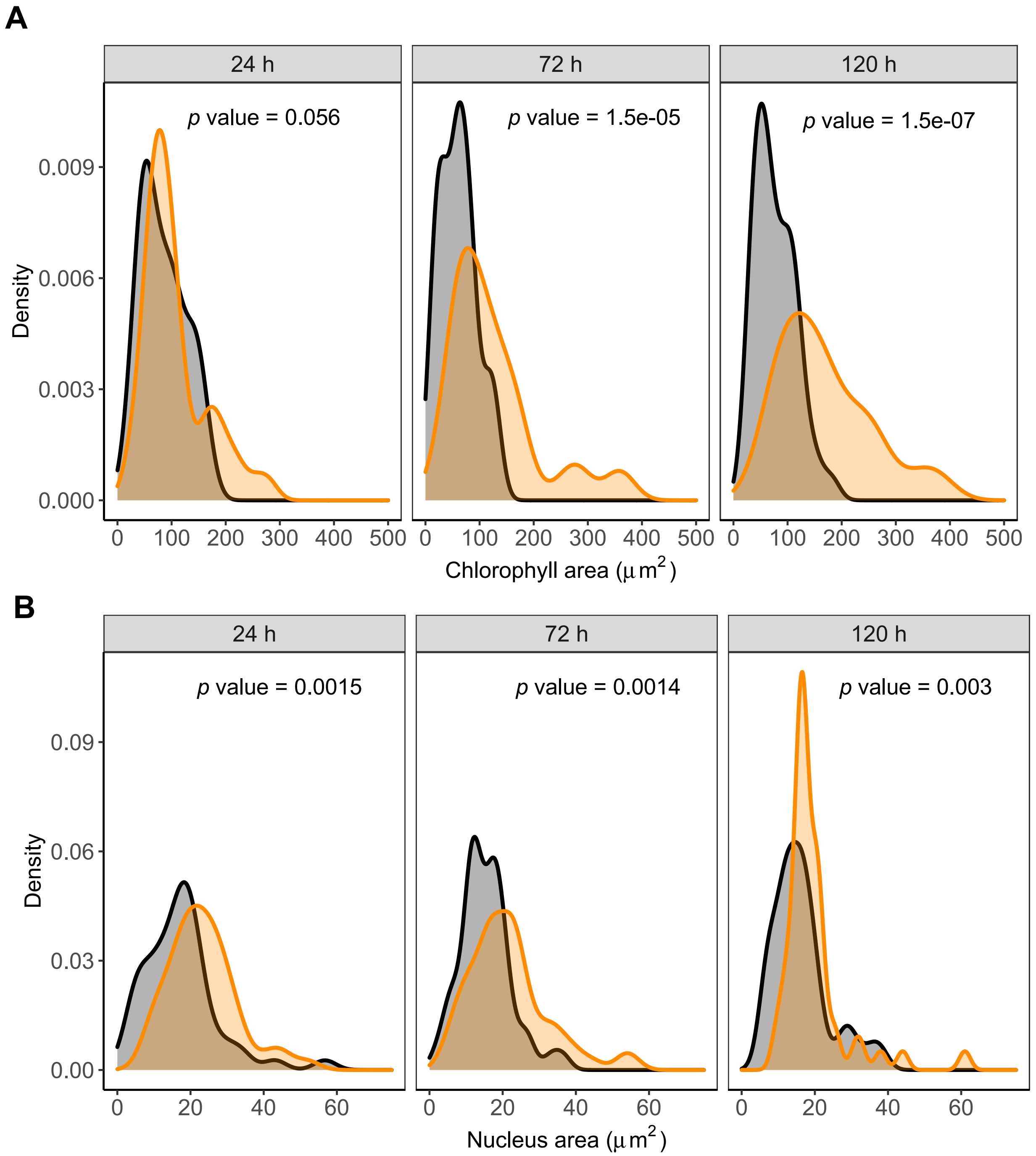

Figure S8. Microscopy measurements of control (black) and *C. atlanticus* filtrate treated (orange) *T. pseudonana* cells. (A, B) Distributions of chlorophyll area (A) and nucleus area (B) per cell in control and *C. atlanticus* filtrate treated cells across three time points. Kolmogorov-Smirnov test was used to test for significance (p<0.05) between control and *C. atlanticus* filtrate treated populations at each time point.

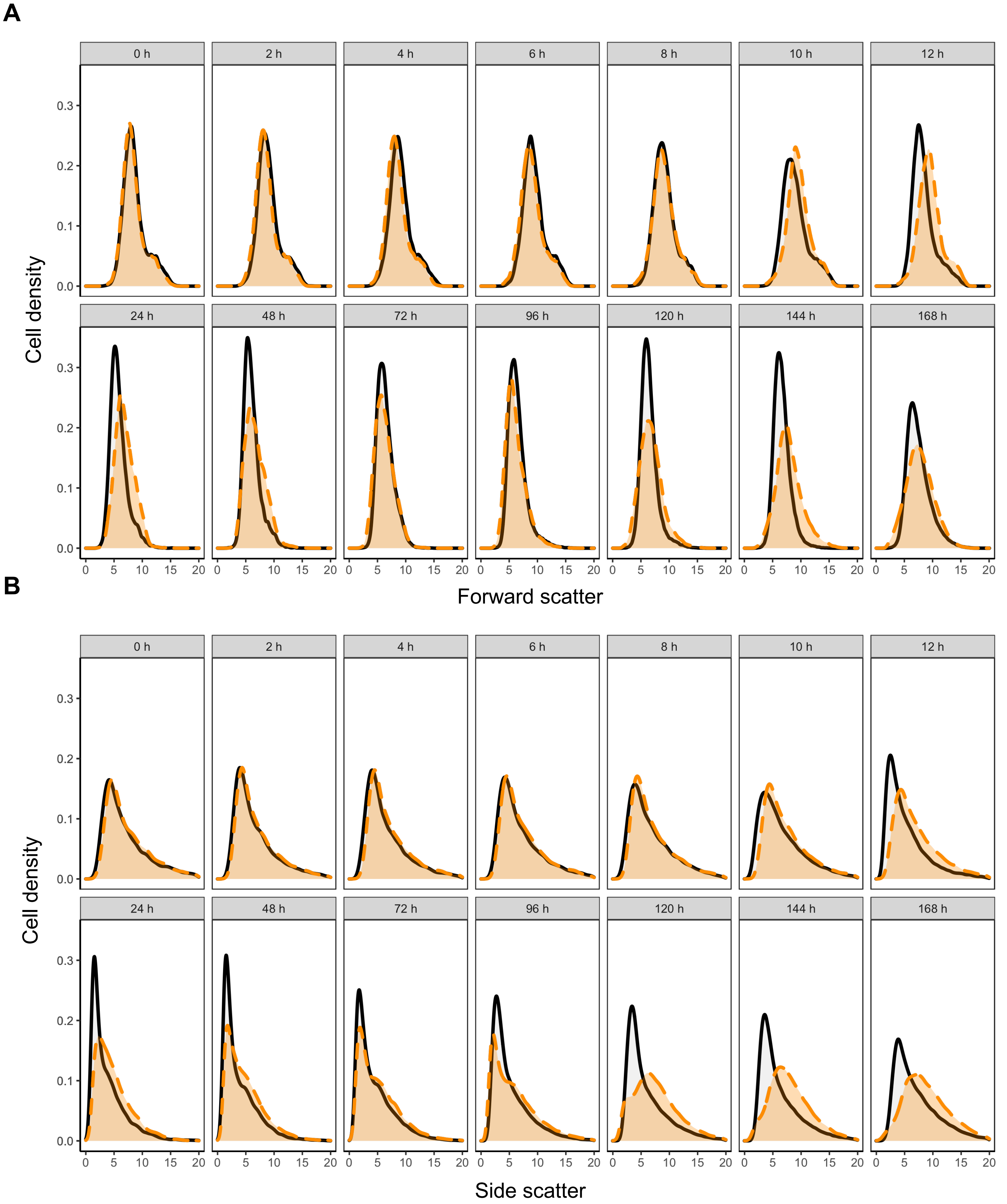

Figure S9. Cell size distributions from flow cytometry data of control (black) and *C. atlanticus* filtrate treated (orange) populations. (A) Distributions of forward scatter as a proxy for cell size. **(B)** Distributions of side scatter can indicate changes in cell shape.

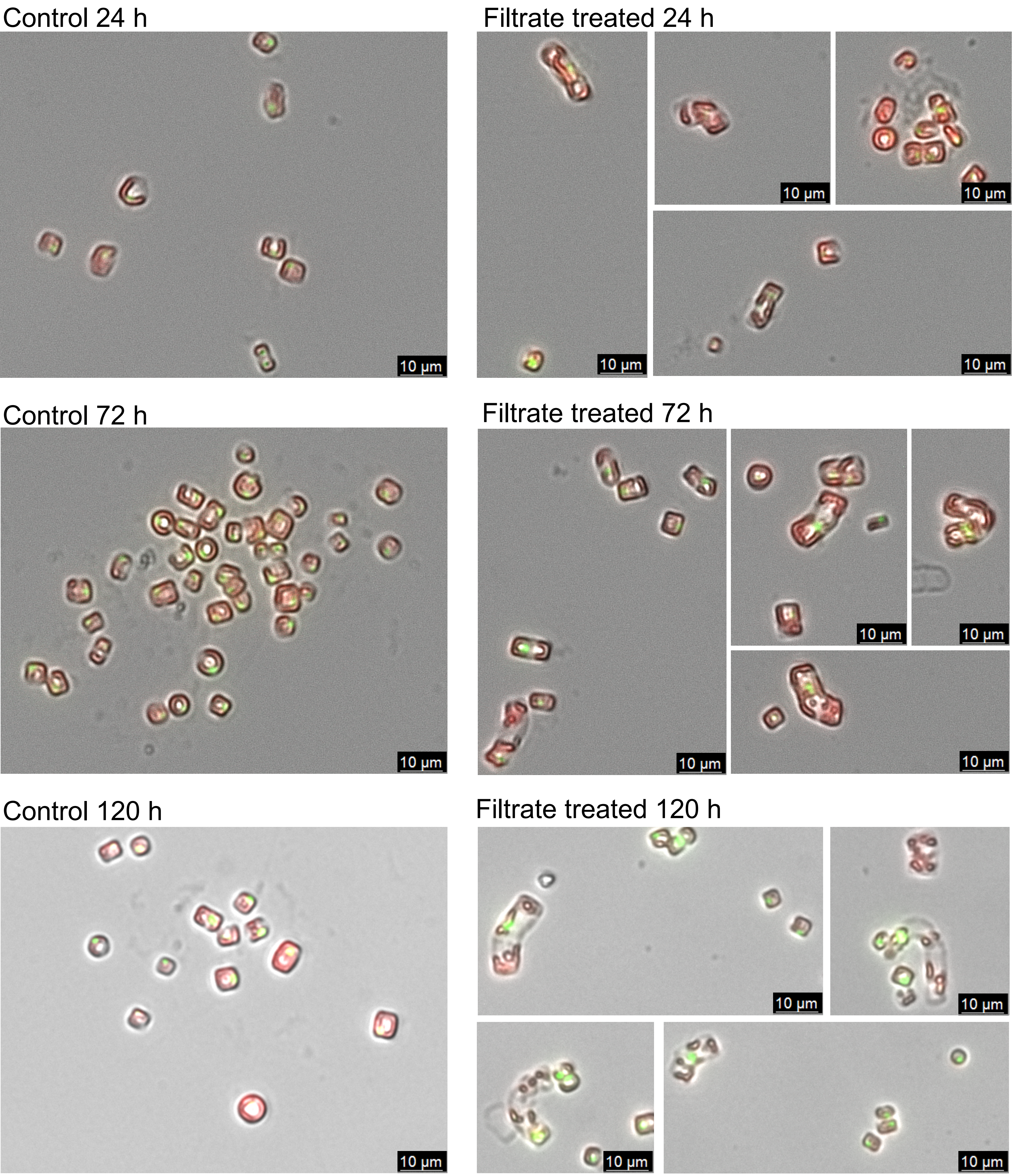

Figure S10: Additional microscopy images of control (left column) and *C. atlanticus* filtrate treated cells (right columns) at three time points. Brightfield images show cell size and shape, overlapped with fluorescence images of SYBR Green stained DNA in diatom nuclei (green fluorescence) and chlorophyll fluorescence (red fluorescence).

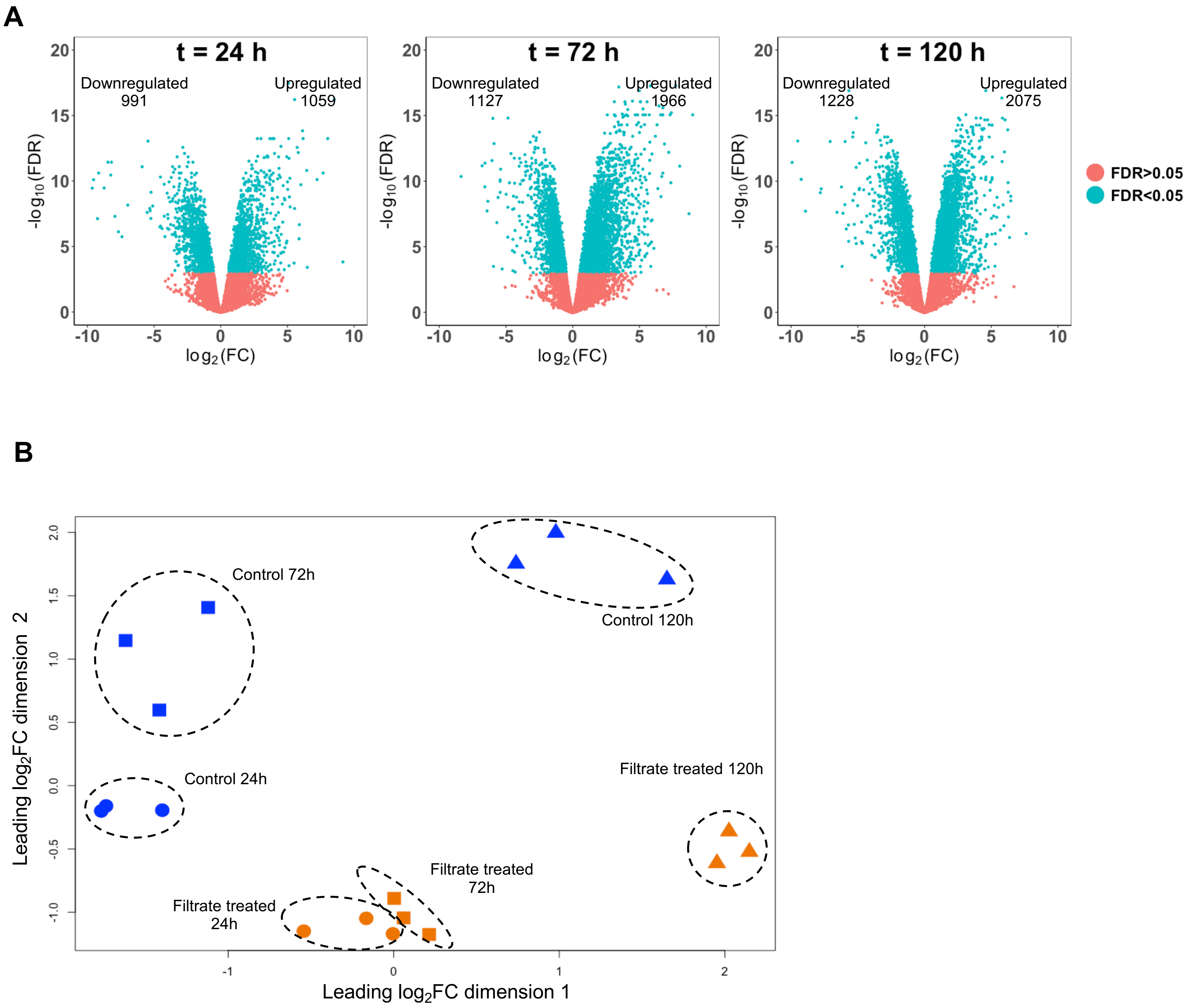

Figure S11. Differential expression analysis of transcriptome data (A) Volcano plots of RNA-seq data for the three sampled time points. Log_2 fold changes are plotted against log_{10} False discover rate (FDR) for *C. atlanticus* filtrate treated samples when compared to the control at all three time points. Significantly differentially expressed genes based on a 5% FDR level are highlighted in blue. The number of significantly up- and down-regulated genes is signified on the plots, where significant differential expression is defined as genes that have $p < 0.01$, FDR < 0.05 and $|log_2$ fold change$| > 1$ in the treatment compared to control samples. **(B)** The Mean Square Deviation (MSD) of the 500 most differentially expressed genes. Different symbols represent samples taken at t = 24 h (circles), t = 72 h (squares) or and t = 120h (triangles) for either *C. atlanticus* filtrate treatment (orange) or control (blue). Distances between samples represent the log_2 fold change between samples, based on the top 500 most discriminative genes.

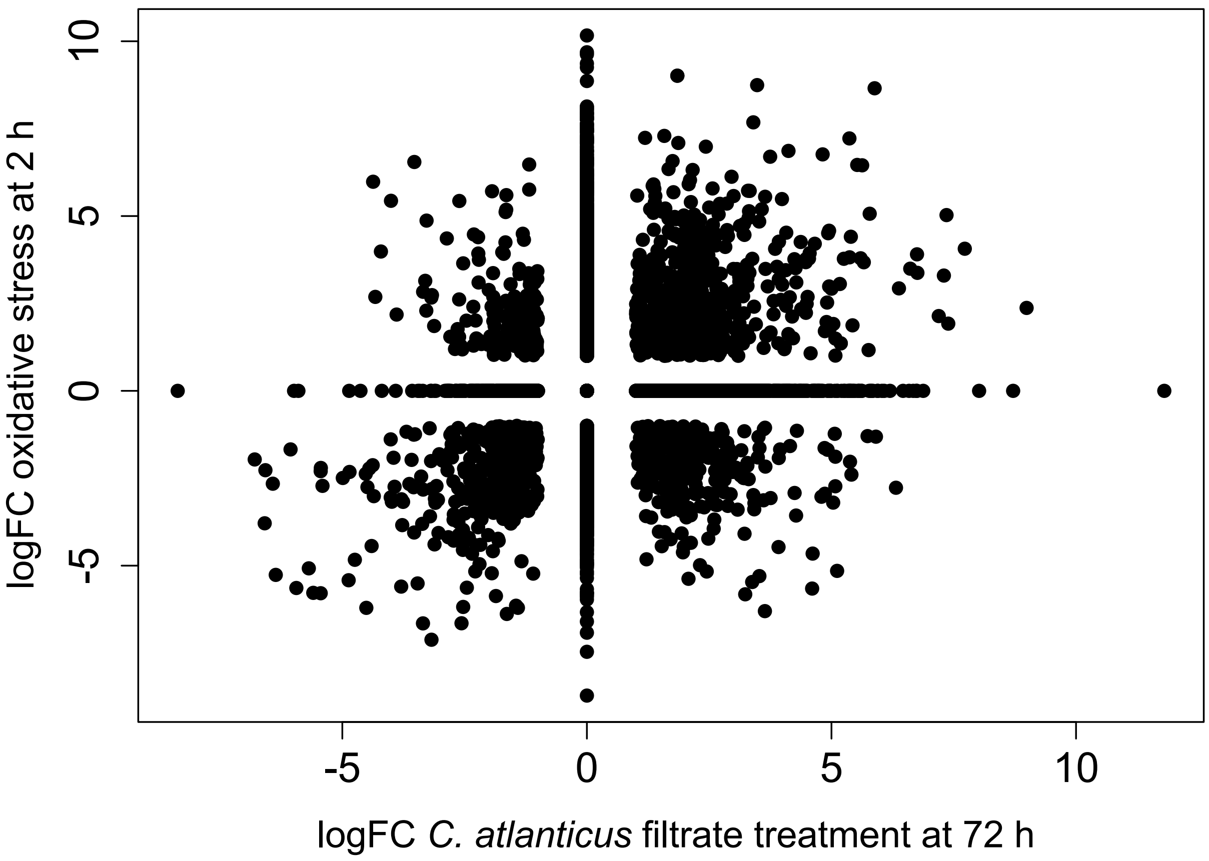

Figure S12. Comparison of *T. pseudonana* transcriptional profiles under *C. atlanticus* filtrate treatment and H_2O_2-induced oxidative stress. Differential expression of *C. atlanticus* filtrate treated after cells after 72 h compared to differential expression 2 h after addition of 200 µM H_2O_2-induced oxidative stress.

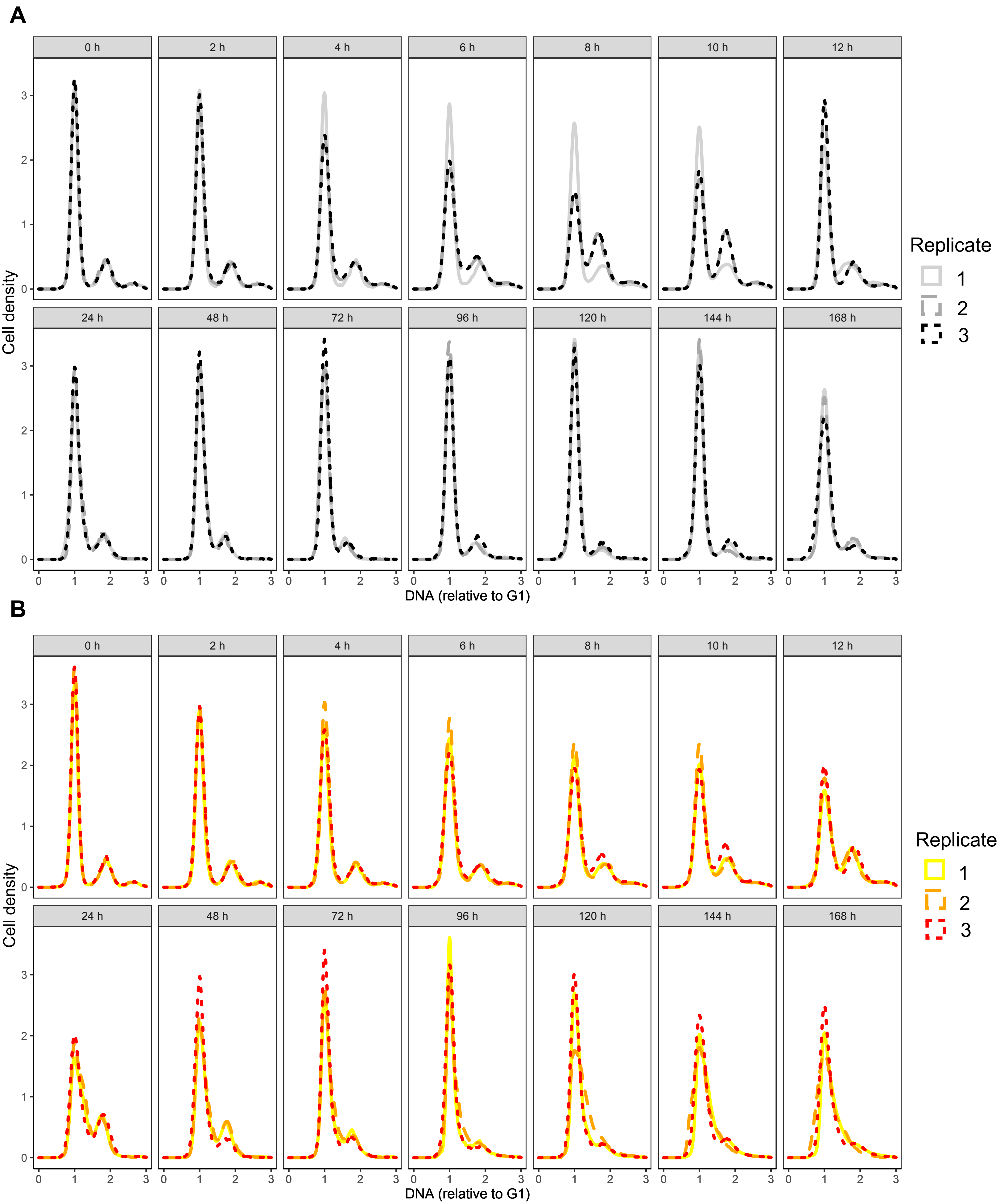

Figure S13: Cell cycle analysis via flow cytometry in control (A) and *C. atlanticus* filtrate treated *T. pseudonana* cells (B). DNA distributions of all cytograms are shown, with distributions normalized to 1 µm beads and aligned based on G1 peaks. For each treatment, all three replicates are plotted to show consistency.

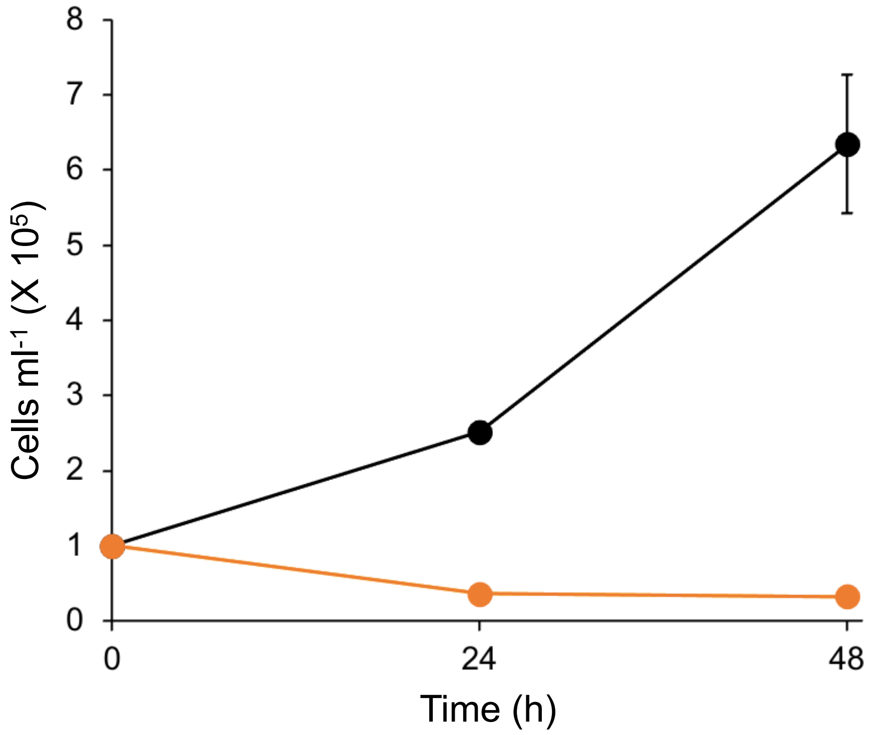

Figure S14: Cell counts of the carbohydrate content phenol-sulfuric acid assay experiment of control (black) and *C. atlanticus* filtrate treated (orange) diatom cells. *T. pseudonana* cells were treated with undiluted bacterial filtrate. At 24 h, cells are still alive, but after 48 h, the undiluted bacterial filtrate treatment becomes lethal.

Supplementary Tables:

All supplementary tables are deposited and available on Zenodo (10.5281/zenodo.6672614).

Supplementary Table 1. Enriched Gene Ontology (GO) terms in WCGNA modules and differentially expressed genes at each of the three time points. Enrichment was done on GO Biological Process (BP), Molecular Function (MF), and Cellular Component (CC) terms, as well as on KEGG pathways on genes belonging to every module and differentially expressed genes at 24 h, 72 h and 120 h after treatment with *C. atlanticus* filtrate. Only significantly enriched terms with Fisher's test p-value < 0.01 are shown.

Supplementary Table 2. Gene significance and module membership for genes in all WGCNA modules. Gene significance (GS), p-value for gene significance (p.GS), module membership (MM) and p-value for module membership (p.MM) for each gene in relation to each measured experimental trait and module.

Supplementary Table 3. Expression patterns of *T. pseudonana* in response to *C. atlanticus* filtrate. Differential expression for three time points and gene significance for *C. atlanticus* filtrate treatment for hub genes with functional annotations, as well as for additional genes involved in relevant pathways and functions that were differentially expressed but were not identified as hub genes.
